# Systematic data capture reduces the need for source data verification: exploratory analysis from a phase 2 multicenter randomized controlled platform trial

**DOI:** 10.1038/s43856-025-01126-9

**Published:** 2025-10-29

**Authors:** Ali B. Abbasi, Kathleen D. Liu, Derek W. Russell, D. Clark Files, Karl W. Thomas, Fady Youssef, Sheetal Gandotra, Andrea Discacciati, Noha Lim, Adam L. Asare, Martin Eklund, Michael Matthay, Laura J. Esserman, I-SPY COVID Investigators, Neil R. Aggarwal, Ellen L. Burnham, Carrie Higgins, Jeff McKeehan, Timothy Albertson, Angela Haczku, Erin Hardy, Richart Harper, Brian Morrissey, Christian Sandrock, Sara Auld, Philip Yang, Joshua Detelich, Gavin Harris, Katherine Nugent, Max Adelman, Jeremy R. Beitler, Anita Darmanian, Amy L. Dzierba, Ivan Garcia, Katarzyna Gosek, Purnema Madahar, Aaron M. Mittel, Justin Muir, Amanda Rosen, John Schicchi, Alexis L. Serra, Romina Wahab, Paul Berger, Carolyn S. Calfee, Melissa Coleman, Alejandra Jauregui, Nathan Cobb, Rajiv Sonti, Amen M. Hamed, Alessio Crippa, Andrea Discacciati, Martin Eklund, Laura Esserman, D. Clark Files, Karl Thomas, Kevin W. Gibbs, Leigha Landreth, Mary LaRose, Lisa Parks, Adina Wynn, Eliot Friedman, Derek W. Russell, Donna Harris, Abhishek Methukupally, Siddharth Patel, Sheetal Gandotra, Kashif Khan, Se Fum Wong, Albert Yen, Jonathan Koff, Lindsie Boerger, John Kazianis, Santhi Kumar, Kathleen D. Liu, Thomas R. Martin, Mark M. Wurfel, Michael A. Matthay, Brian Daniel, Nuala J. Meyer, Caroline A. G. Ittner, Nilam S. Mangalmurti, John P. Reilly, Timothy Obermiller, Philip A. Robinson, Farjad Sarafian, Usman Shah, Richard G. Wunderink, G. R. Scott Budinger, Helen K. Donnelly, Benjamin D. Singer, Fady A. Youssef, Daniel Belvins, Catherine Nguyen, Alexis Suarez, Maged A. Tanios, Scott Fields, James Hurst-Hopf, Lamorna Brown Swigart, Christina Creel-Bulos, Christina Spainhour, Ari Moskowitz, Praveen Vijhani, Adrienne M. Casciato, Vaney Capetillo, Kenenth Wei, Tracie Huynh, Anna Rodriguez-Vasquez, Joseph L. Nates, Jessica Suarez, Siddharth Nair, Sandya Samavedam, Michael Bernstein, Christopher Jordan, Daniel H. Kett, Karla Leon Escalona, Richard A. Lee, Kenneth Remy, Ali Zarrinpar, Robert Hyzy, Kristine Nelson, Caroline Quill, Emilio Mazza, Kristin Broderick, Jermiah Hayanga, Ana Costa Monteiro, Joseph Levitt, Ruixiao Lu, Paul Henderson, Adam Asare, Imogene Dunn, Alejandro Botello Barragan

**Affiliations:** 1 University of California San Francisco, San Francisco, CA USA; 2 University of Alabama at Birmingham, Birmingham, AL USA; 3 Wake Forest University School of Medicine, Winston-Salem, NC USA; 4 MemorialCare Long Beach Medical Center, Long Beach, CA USA; 5 Karolinska Instituet, Stockholm, Sweden; 6 Quantum Leap Healthcare Collaborative, San Francisco, CA USA; 7 Pulmonary Sciences & Critical Care, University of Colorado, Aurora, CO USA; 8 Division of Pulmonary, Critical Care and Sleep Medicine, University of California Davis, Sacramento, CA USA; 9 Department of Medicine, Emory University, Atlanta, GA USA; 10 Centre for Acute Respiratory Failure, Columbia University, New York, NY USA; 11 Department of Critical Care, Sanford Health, Sioux Falls, SD USA; 12 Division of Pulmonary, Critical Care, Allergy and Sleep Medicine, University of California San Francisco, San Francisco, CA USA; 13 Pulmonary and Critical Care Medicine, Georgetown University, Washington, DC USA; 14 Department of Medical Epidemiology and Biostatistics, Karolinska Institutet, Solna, Sweden; 15 Department of Surgery, University of California San Francisco, San Francisco, CA USA; 16 Pulmonary and Critical Care Medicine, Wake Forest University, Winston-Salem, NC USA; 17 Department of Medicine, Main Line Health, Wynnewood, PA USA; 18 Pulmonary, Allergy, and Critical Care Medicine, University of Alabama Birmingham, Birmingham, AL USA; 19 Pulmonary Critical Care, University of Maryland Medical Center, Baltimore, MD USA; 20 Critical Care Medicine, Keck School of Medicine University of Southern California, Los Angeles, CA USA; 21 Pulmonary, Critical Care, and Sleep Medicine, Yale University, New Haven, CT USA; 22 Division of Pulmonary, Critical Care and Sleep Medicine, Keck School of Medicine University of Southern California, Los Angeles, CA USA; 23 Divisions of Nephrology and Critical Care Medicine, University of California San Francisco, San Francisco, CA USA; 24 Pulmonary, Critical Care and Sleep Medicine, University of Washington, Seattle, WA USA; 25 Critical Care Medicine, University of California San Francisco, San Francisco, CA USA; 26 Department of Medicine, University of Pennsylvania Perelman School of Medicine, Philadelphia, PA USA; 27 Critical Care Medicine, Logan Health Research Institute, Kalispell, MT USA; 28 Infectious Disease, Hoag Medical Group, Newport Beach, CA USA; 29 Department of Medicine, Pulmonary and Critical Care Division, Northwestern University Feinberg School of Medicine, Chicago, IL USA; 30 Department of Internal Medicine, Long Beach Memorial Medical Centre, Long Beach, CA USA; 31 Investigational Drug Service, University of California San Francisco, San Francisco, CA USA; 32 Department of Laboratory Medicine, University of California San Francisco, San Francisco, CA USA; 33 Department of Anesthesiology, Emory University, Atlanta, GA USA; 34 Department of Emergency Medicine, Emory University, Atlanta, GA USA; 35 Division of Critical Care Medicine, Montefiore Medical Centre, Bronx, NY USA; 36 Pulmonary and Critical Care Medicine, DHR Health, Edinburg, TX USA; 37 Kaiser Permanente Los Angeles Medical Center, Los Angeles, CA USA; 38 Department of Critical Care Medicine, Division of Anesthesiology, Critical Care Medicine and Pain Medicine, MD Anderson Cancer Center, Houston, TX USA; 39 Critical Care Medicine, Mercy Hospital, Springfield, MO USA; 40 Critical Care Medicine, Stamford Health, Stamford, CT USA; 41 Critical Care Medicine, University of Miami, Miami, FL USA; 42 Pulmonary and Critical Care Medicine, University of California Irvine, Irvine, CA USA; 43 Critical Care Medicine, Innovations in Pulmonology, Critical Care & Sleep Medicine, Cleveland, OH USA; 44 Division of Transplantation and Hepatobiliary Surgery, University of Florida, Gainesville, FL USA; 45 Critical Care Medicine, University of Michigan, Ann Arbor, MI USA; 46 Division of Pulmonary & Critical Care Medicine, University of Rochester, Rochester, MN USA; 47 Pulmonary, Critical Care and Sleep Medicine, Virtua Our Lady of Lourdes Hospital, Evesham, NJ USA; 48 Surgical Critical Care, West Virginia University, Morgantown, WV USA; 49 Pulmonary and Critical Care, University of California Los Angeles, Los Angeles, CA USA; 50 Pulmonary, Allergy and Critical Care Medicine, Stanford University, Stanford, CA USA

**Keywords:** Clinical trials, Drug development

## Abstract

**Background:**

The COVID-19 pandemic gave rise to clinical trials focused on systematic, accurate primary data capture, and reduced reliance on source data verification (SDV). Here, we report on a natural experiment that allowed us to assess the quality, cost, and impact of this approach compared to traditional SDV.

**Methods:**

The I-SPY COVID trial (NCT04488081) was a multicenter, open-label, platform trial that employed a streamlined daily checklist, daily capture of labs and medications, and centralized monitoring to ensure accurate data collection in lieu of SDV. The trial enrolled 1,111 patients in 11 drug arms with severe COVID-19. After the trial arms were closed, extensive retrospective SDV was performed on 333 (30.0%) patients, including 10,101 of 44,486 (23%) electronic case report forms (eCRFs), allowing us to evaluate the impact of our strategy on data integrity, outcomes, and costs.

**Results:**

We find that retrospective SDV results in changes to 0.36% (1,234 / 340,532) of data fields. It results in no changes to the type of outcome recorded (death, recovery, or censored), but changes in the day of recovery in 9 instances, by a median of 2 days (range 1-7). Two additional AEs are added during SDV that had not previously been captured. Costs associated with retrospective SDV of 23% eCRFs are 61,073 person-hours at a cost of $6.1 M.

**Conclusions:**

Extensive SDV does not change any results or conclusions of the I-SPY COVID trial, which was designed with a systematic strategy for data capture, monitoring, and safety. This strategy could improve the efficiency of clinical trials and eliminate the need for manual SDV.

## Plain Language Summary

This study aimed to determine whether using a systematic approach to record clinical trial data accurately in the first place would reduce the need to re-check the data for errors later. The approach combined automated transfer of laboratory data, simplified electronic forms for clinical trial staff to complete and ongoing monitoring for safety by a committee of physicians. Re-checking the data after the trial had completed, known as “source data verification” or SDV, revealed very few errors and none that changed the result or interpretation of the clinical trial’s findings. Because SDV is very time consuming and costly, this study may provide a way to help reduce the overall costs of clinical trials.

## Abstract (web summary)

Abbasi et al. evaluate the value of source data verification, a costly but traditional element of clinical trials. They find it is largely redundant when used with a systematic strategy to promote efficient and accurate data capture, monitoring, and safety in a phase II platform trial.

**Trial Registration**: https://clinicaltrials.gov/study/NCT04488081

## Introduction

Randomized clinical trials (RCTs) are essential to test the efficacy and safety of treatments, but the time, administrative effort, and cost required to run these trials are substantial and rising steadily, threatening the productivity of the clinical trials enterprise^1,2^. One of the major contributors to trial costs is monitoring, which ensures the trial is conducted in accordance with the protocol and that data are entered accurately and completely. Traditionally, the latter is accomplished using source data verification (SDV) of the data housed in the electronic data capture system (EDC) by comparing it with source data in the electronic health record (EHR)^3,4^. While SDV serves a valuable purpose, it has become one of the more time-consuming and expensive trial processes, accounting for up to 30% of trial costs^3,5^.

The COVID-19 pandemic and its associated restrictions presented numerous challenges to the usual conduct of clinical trials, including monitoring, as site visits by study monitors were not possible. This was particularly problematic for early therapeutic trials in COVID, which were desperately needed at the time. These conditions led to the increased adoption of innovative trial designs that included approaches to capture data accurately in the first place rather than verifying the accuracy of the data through monitoring with SDV^6^. However, it is not clear how these innovative trial designs impacted the fidelity of data capture and participant safety assurance.

Here, we report an analysis of data captured in a large, multicenter platform trial, I-SPY COVID, that employed an innovative monitoring and safety strategy. I-SPY COVID was designed to rapidly test agents for large improvements in odds of recovery or death in severe COVID-19^7^. A focused data capture, monitoring, and safety strategy was designed to maximize accuracy of primary data capture in critically ill patients without reliance on SDV, by deploying a short daily checklist for each patient, daily recording or automated capture of labs, and an innovative centralized monitoring strategy. Relying on these approaches, we performed no in-person monitoring or SDV while the trial was enrolling patients.

However, as the pandemic eased, one of the trial’s funders required us to conduct extensive retrospective SDV, which was initiated after all trial arms had been closed. This created a natural experiment to evaluate the impact of I-SPY COVID’s innovative data capture, monitoring, and safety strategy on data integrity, including the recording of adverse events (AEs) and the primary outcome (time to recovery and mortality), compared to the traditional strategy of extensive SDV. In this retrospective analysis, we find that a focused data capture, monitoring, and safety strategy can vastly reduce the need for extensive SDV and implementing such a strategy can result in very substantial labor and cost savings.

## Methods

### Trial design

The I-SPY COVID trial is a phase II open-label multicenter randomized platform trial for patients hospitalized with COVID-19 who require high-flow oxygen or intubation (clinicaltrials.gov/study/NCT04488081). Many of the design elements were adapted from the I-SPY2 cancer trial^8,9^, and the trial protocol has been published^7^. Briefly, the trial randomized patients concurrently into one of several investigational agent arms or a control group. The co-primary outcomes were death and time to recovery, defined as improvement to WHO COVID level ≤4^10^ for at least 2 consecutive days^10^. The trial included 42 clinical sites (30 master sites and 12 satellites) in the U.S. and was approved by a central Institutional Review Board (IRB) (Wake Forest University IRB, Winston-Salem, NC). I-SPY COVID was conducted in accordance with Good Clinical Practice guidelines and the Helsinki Declaration, and informed consent was obtained for all participants prior to enrollment in the interventional arms of the study. For patients unable to provide informed consent due to a lack of adequate decision-making capacity, a legally authorized representative was identified for the provision of prospective consent as required by the policies of the overseeing institutional review board and all relevant local institutions.

The trial was designed and implemented by the trial sponsor, Quantum Leap Healthcare Collaborative. The sponsor was also responsible for centralized monitoring of the trial, including site compliance with the protocol, and centralized safety monitoring. The trial was funded by a consortium involving industry, non-profit organizations, and federal agencies. With the exception listed in this section, trial funders had no role in designing the trial.

### Data capture

**Fig. 1:**
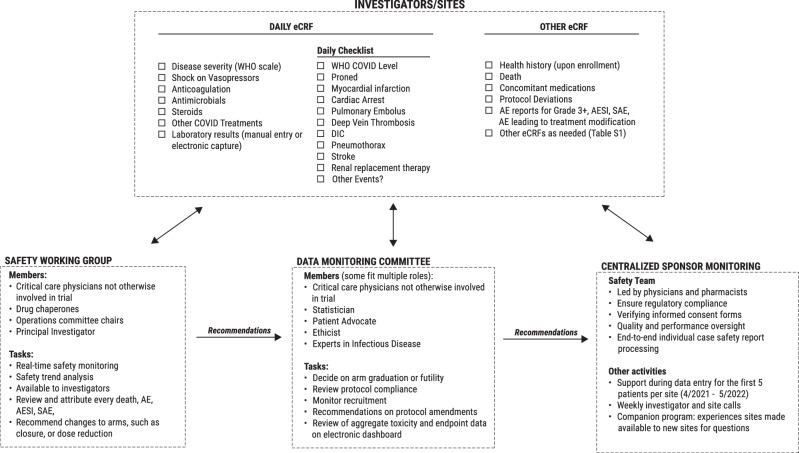
Data collection and monitoring in the I-SPY COVID trial. Data collection was systematic and standardized by deploying a short daily checklist and daily entry or capture of concomitant medications and labs. Data from the sites were available to the safety working group (SWG), the data monitoring committee (DMC), and the sponsor. The SWG performed real-time, centralized safety monitoring and was available to investigators to assist with managing the study agents. The SWG provided recommendations on the management of the agents to the DMC. The DMC was provided with an electronic dashboard that enabled centralized review of aggregate toxicity and endpoint data, and made recommendations to the sponsor on safety, graduation, and futility of the study agents.

Data capture in I-SPY COVID was optimized to minimize the burden on clinicians, while capturing essential data elements accurately and completely. The core data collection tool was the daily eCRF, which included details on disease severity, treatments received, concomitant medications, and a discrete set of labs to enable evaluation of all major organ systems. A key innovation was that the daily eCRF also included a short checklist of important clinical events that were predefined by trial stakeholders (Fig. 1 and Supplementary Fig. 1). The checklist required sites to assess daily whether the patient suffered AEs common in critical illness due to COVID-19, such as a pneumothorax, pulmonary embolism, or initiation of antibiotics. The goal of this checklist was to limit reporting bias in this open-label trial by capturing AE data systematically on all participants.

At the start of the trial, concomitant medications and labs were entered manually by trial coordinators or investigators, but over time, we implemented automated transfer of these data from the EHR to the EDC, known as electronic source data capture, using the OneSource data capture platform^11^. OneSource uses Fast Healthcare Interoperability Resources (FHIR^®^)-based application programming interfaces (APIs) to automatically extract EHR data. FHIR APIs are electronic data access portals that allow access to certain data fields with standardized requests, instead of needing to customize data requests for different EHR installations. FHIR APIs are widely available in the U.S. because they are a requirement for federal EHR certification^12^. For lab results, OneSource included automatic capture of reference ranges, which enabled automatic grading of lab AEs. Full implementation of OneSource was achieved at 15 sites by the end of the trial.

The daily eCRF was designed and structured so that AEs entered were automatically graded using the Common Terminology Criteria for AEs (CTCAE) v5.0. For laboratory results, data were normalized according to the reference range at each institution, dividing the lab result by the upper limit of the reference range. Regardless of whether AEs were already recorded in the checklist on the daily eCRF, investigators were also asked to complete dedicated AE electronic Case Report Forms (AE eCRFs) for any unanticipated AEs, AEs grade ≥3 attributed to study agents, serious AEs (SAEs), and AEs of special interest (AESI). AESI were pre-specified for each agent and reported both for the drug arm and concurrent controls (Table 1).

**Table 1:** List of study agents

Agent name*	Date arm closed	*N*	Mechanism	AE of special interest
Remdesivir/Corticosteroid	Backbone	NA	Protide/Immunomodulator	Abnormal liver function
Razuprotafib (Aerpio)	2021.03.12	22	Vascular endothelial PTP inhibitor/ activator of Tie-2	None
Apremalist (Otezla)	2021.03.05	68	PDE4 inhibitor	None
Cenicriviroc	2021.04.28	91	CCR2 and CCR5 inhibitor	Abnormal liver function
Icatibant/Firazyr	2021.04.15	96	Bradykinin inhibitor	None
Pulmozyme	2022.01.10	39	Recombinant DNAse	Abnormal liver function, transmission of infectious agent via study drug
IC14	2021.12.03	67	Anti-CD14 antibody	New infections, Eye symptoms
Famotidine + Celecoxib	2021.09.02	30	Famotidine: H2 receptor antagonist and inverse agonist Celecoxib: COX-2 inhibitor, NSAID	Famotidine:Thrombocytopenia, sustained ventricular arrythmia Celecoxib: cardiovascular thrombotic events, GI bleeding, heart failure, acute kidney injury, anaphylaxis, Stevens-Johnson syndrome
Narsoplimab	2022.09.15	68	IgG4 against MASP-2, an effector enzyme of the lectin pathway of complement	Infusion reactions, anaphylaxis, elevated liver enzymes, infections
Aviptadil	2021.06.15	52	Synthetic human vasoactive intestinal peptide	Diarrhea (Grade 3+)
Cyclosporine	2022.08.08	110	Calcineurin inhibitor	Infection, hypertension, neurotoxicity, acute kidney injury, abnormal liver function
Cyproheptadine	2023.01.04	35	Antihistamine and serotonin antagonist with anticholinergic effects	Delirium or hallucinations, urinary retention, mucous obstruction, ilieus

* Note that all patients also received backbone therapy consisting of Remdesivir and a corticosteroid. *PTP* protein tyrosine phosphatase, *PDE* phosphodiesterase, *CCR* C–C chemokine receptor, *NSAID* nonsteroidal anti-inflammatory, *MaSp* Mannan-binding lectin-associated serine protease-2; *Tie-2* tyrosine kinase with immunoglobulin-like and epidermal growth factor (EGF)-like domains 2; *IgG* Immunoglobulin G.

### Centralized monitoring and safety strategy

To ensure protocol compliance, data fidelity, and a high standard of safety monitoring in a critically ill population despite the limitations imposed by the pandemic, centralized monitoring and review were implemented, relying on the safety working group (SWG), the Data Monitoring Committee (DMC), and the sponsor’s in-house staff (Fig. 1).

The Safety Working Group (SWG) was a committee of intensive care/critical care physicians in active academic clinical practice, which replaced and expanded on the function of the medical monitor commonly used in clinical trials. The SWG was co-chaired by 2 independent investigators not otherwise involved in the trial and included representation by 1 trial PI (KL), 2 ‘drug chaperones’ for active agents (the lead investigators for each arm), and a safety officer from the sponsor (Fig. 1). The SWG met for about 2 h per week throughout the duration of the trial to examine all deaths, SAEs, AESIs, and AEs grade ≥3 that the investigator judged to be possibly, probably, or definitely attributable to a study drug. The SWG facilitated rapid review of clinical events to enable final adjudication and attribution for any SAEs and AESIs. Unlike a traditional medical monitor, the SWG was more actively involved in the trial by helping manage unexpected AEs. For example, the SWG (in collaboration with the DMC) could recommend dose changes or additional safety monitoring requirements to manage emerging toxicities related to the study drugs. The SWG also made recommendations to the DMC regarding safety concerns or discontinuation of study drugs at the completion of a safety run-in or dose modifications. The sponsor provided partial salary support of about $21,500 per annum for the 2 SWG chairs, but not the investigators or drug chaperones.

The DMC took the role of the traditional independent Data Safety and Monitoring Board and was chaired by external critical care physicians and experienced trialists who met biweekly to review study data, including outcomes assessment and safety parameters. The DMC reviewed study data, alongside reports and recommendations from the SWG, to make recommendations regarding the termination of study arms, reviewed reports of protocol deviations, monitored recruitment, and made recommendations regarding protocol amendments. To facilitate efficient review, the DMC was given access to an online dashboard that provided an overview of the trial, including enrollment progress, listings of AEs, and an automated comparison of lab values (using automated CTCAE grading as described above) and clinical events collected on the checklist between active arms and control arms (see Supplementary Fig. 2).

The sponsor also performed centralized monitoring, which was facilitated by the sponsor’s in-house safety team of experienced professionals in pharmacovigilance, consisting of three individual contributors and one manager/director serving the I-SPY COVID trial and other platform trials. This group ensured compliance with regulatory requirements, for example, by verifying that informed consent forms had been completed, reviewing data quality, processing individual safety reports, and hosting meetings of the SWG. The sponsor also implemented additional programs to help sites, including weekly calls with investigators and sites, and a companion program allowing more experienced sites to be available for questions from new sites.

### Source data verification (SDV)

Most clinical trials prior to the COVID-19 pandemic used in-person monitoring and SDV to ensure the accuracy and completeness of trial data, which required monitors to visit the trial sites in real time to compare the data in the eCRFs to original data sources such as the EHR. In I-SPY COVID, due to pandemic-related limitations, no in-person sponsor monitoring or SDV was performed while the trial arms were open from July 2020 through May 2022. However, starting in April 2021, we instituted a process whereby one of the senior experienced clinical research coordinators was assigned to work with each new site on data entry of the first five patients accrued on the trial to ensure fidelity to the new process. This support was conducted over video conferencing and focused on facilitating training of site staff to become familiar with the trial data entry procedures, rather than directly verifying source data.

Subsequently, a federal funder, the Biomedical Advanced Research and Development Authority (BARDA), requested extensive retrospective monitoring and SDV to align the study with more traditional monitoring strategies. As a result, retrospective, in-person monitoring, including SDV, was performed between 6/1/2022–12/31/2022, after all 11 arms had been terminated. Initially, while developing a risk-based monitoring (RBM) plan agreeable to the funder, we started SDV across all sites, selecting patients for SDV at the discretion of the sites. During this period, the initial goal was to perform 100% SDV on the selected patients, but before this process was completed, we transitioned to an RBM plan. At the time of transition to RBM, we had performed partial SDV of eCRFs relating to 324 patients. Once the funder agreed to our RBM plan, we transitioned to targeted monitoring of at least 10% of all subjects or 2 subjects per site, whichever was greater, and focused monitoring only on medium and high-risk eCRFs (Supplementary Table 1). These participants were chosen at the discretion of the sites and monitors, and no formal guidance was provided for participant selection. This period of RBM included 137 patients, most of whom had already undergone some SDV during the initial phase of SDV. Due to this overlap, the total number of patients that underwent any retrospective SDV of eCRFs was 333.

### Evaluating the accuracy of data capture with retrospective SDV

We counted the number of eCRFs and data fields subject to SDV and the number of fields changed during the retrospective SDV period (6/1/2022 − 12/31/2022), and compared the number of changes by site and eCRF. To understand the impact of electronic source data capture on data fidelity, we also compared the rate of change in data fields after the implementation of OneSource, compared to before, using a chi-squared test.

Because trial activities were happening concurrently (e.g., SWG and DMC meetings, data cleaning by the sites), not all changes in the EDC during this period were the result of retrospective SDV. To determine which changes in the primary outcome and AEs were related specifically to the retrospective SDV process, we performed the following adjudication process: For co-primary outcomes, we identified which participants had changes in the primary endpoint during the period of retrospective SDV and captured the EDC metadata associated with these changes (e.g. annotations, queries, reasons for change, audit logs, and monitoring visit reports). Changes to a record were recorded as unrelated to SDV if no SDV was noted in the metadata. If SDV had occurred, we checked for explicit documentation indicating the change resulted from SDV. If metadata was inconclusive, we adjudicated on a case-by-case basis, based on a comprehensive review of the metadata, the date of SDV, and the data change date (Supplementary Fig. 4). For AEs, we identified any new AE entered during the retrospective SDV period. If the patient had undergone SDV, we searched the EDC for related queries to determine the source of the change. To evaluate the impact of SDV on the overall trial conclusions, we compared arm termination reports that were generated prior to the start of SDV to those that were generated after the conclusion of SDV (see Supplementary Methods).

### Cost of retrospective monitoring

We quantified the cost and time required to conduct retrospective monitoring both for the sponsor and the sites. The total cost and time required for monitoring were calculated by summing the cost and time required by the sponsor and by the sites. We calculated the cost per data change by dividing the total cost of retrospective monitoring and SDV by the total number of data changes.

The sponsor reviewed its personnel and accounting records to estimate the cost and time required for monitoring. The number of hours was estimated by reviewing personnel records and counting the number of full-time equivalent permanent and temporary staff required, and the number of days these staff were engaged in the retrospective monitoring. The sponsors estimated costs associated with monitoring by reviewing accounting records to estimate the total cost associated with the staff involved in retrospective monitoring, including overhead expenses. We estimated the hourly cost of monitoring by dividing the total cost by the total number of staff hours.

To estimate the additional time and cost associated with retrospective monitoring for the sites, we conducted a survey where we asked each site to estimate the number of hours required for remote monitoring visits, in-person visits, onboarding of monitors, and responding to queries (the survey is in Supplementary Table 2). Details of the calculation of the time required to respond to queries are in the Supplementary Methods. The cost to the sites was estimated by multiplying the number of hours by the hourly cost of monitoring, derived as described above.

### Reporting summary

Further information on research design is available in the Nature Portfolio Reporting Summary linked to this article.

## Results

### Description and characteristics of study data

Here, we include results related to all 11 investigational agent arms and the control arm open between 7/30/2020–1/26/2022 (see Table 1), enrolling a total of 1,111 participants across 30 master sites, generating a total of 44,486 eCRFs^13^. The razuprotafib arm was closed after a safety run-in designed by the sponsor because, although the agent was likely safe, it was determined that this arm would require additional safety monitoring that would consume excessive resources early in the pandemic. The remaining 10 arms were closed after meeting pre-defined conditions of futility for demonstrating marked improvement in mortality or recovery^7,13^.

### Data errors identified by retrospective SDV

At the request of one of the study funders, retrospective SDV was performed after the trial arms had closed. Among 1111 patients, 333 (30.0%) underwent any retrospective SDV between 6/1/2022 and 12/31/2022. In terms of eCRFs, 10,101 of 44,486 (23%) underwent SDV, representing a total of 340,532 unique data fields.

**Fig. 2:**
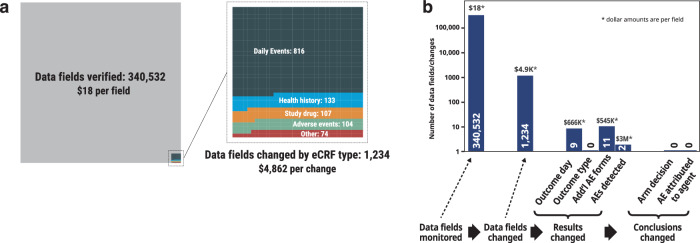
Results and costs of source data verification. **a** Data fields verified, and data fields changed. The chart area corresponds to the number of data fields. **b** Data fields verified, data fields changed, changes to results, and changes to conclusions, with associated costs per event, which were derived by dividing the total cost of monitoring by the number of changes in each category. eCRF electronic case report form, AE adverse event.

The retrospective SDV discovered errors in 1234 (0.36%) data fields (Supplementary Table 3). The highest number of errors was in the daily events eCRF, which was completed by sites daily on each patient (816 errors, 0.4%), health history (133 errors, 1.3%), study drug administration (107 errors, 0.9%), and AEs (104 errors, 0.7%). (Fig. 2a, Supplementary Table 3).

There was also some variation across sites, with data field error rates ranging from 0.0% to 1.2% (Supplementary Table 4, Supplementary Fig. 4). At sites that deployed electronic source data capture using OneSource at any point in the trial, the rate of data errors was lower after the activation of OneSource, compared to before (0.29% vs. 0.37%, chi-squared *p* = 0.03, Supplementary Table 5).

### Resulting changes in primary outcomes

Errors in raw EDC data during retrospective SDV resulted in 25 changes in variables used for assessment of the co-primary outcome. According to the adjudication, 9 of these were discovered as part of retrospective SDV (Table 2), while 16 were discovered during routine Safety Working Group (SWG), Data Monitoring Committee (DMC), or site activity. All SDV-related changes in primary outcomes fields were changes in the day of recovery, and the median magnitude of the change was 2 days (range 1–7). There were no changes in the type of outcome that occurred or the timing of outcomes other than days to recovery. None of the changes in the primary outcome resulted in any arm being reclassified with respect to whether or not the arm met the prespecified efficacy or safety criteria for arm graduation (success) or termination (Supplementary Results, Supplementary Fig. 5).

**Table 2:** Changes in the primary outcome due to monitoring

Patient	Arm	Outcome	Day (pre-monitoring)	Day (post-monitoring)
1	Narsoplimab	Recovered	3	7
2	Cyclosporine	Recovered	3	5
3	Control	Recovered	11	10
4	Cyproheptadine	Recovered	16	17
5	Control	Recovered	6	5
6	Control*	Recovered	11	4
7	Control	Recovered	6	5
8	Control	Recovered	44	48
9	Control	Recovered	3	5

Changes in the primary outcome, recovery or death, that were due to retrospective monitoring. All changes were in the day of recovery. There were no changes in the type of outcome that occurred, and no changes amongst patients who had an outcome other than recovered.

* This was due to a monitoring query of the daily form, which changed to reflect a lower level of oxygen support. As a result, the WHO COVID level was corrected to 4 on days 4 and 5, meeting the definition for recovery. However, on day 6 the patient’s WHO COVID level increased to 5 and remained there until day 11.

### Changes in AEs captured

A total of 398 AEs were recorded in the trial, including 198 amongst the participants who underwent any SDV. Our review of the EDC identified 19 changes in AE grade made during the retrospective SDV period. However, according to our adjudication process, none of these occurred as a result of retrospective SDV and were instead identified by the sites themselves or queries from the sponsor. We also identified 40 new AE eCRFs entered during the period of retrospective SDV. According to our adjudication, 11 of these were created as the result of retrospective SDV (Supplementary Table 6). These included the creation of 6 AE eCRFs relating to laboratory values, which were already captured in the daily labs eCRF, and 3 events that had already been captured in the checklist on the daily events eCRF (Table 3). There were 2 AE eCRFs completed as a result of SDV that had not previously been recorded anywhere else in the EDC, including a grade 1 myocardial infarction and a grade 3 gastrointestinal bleed. Neither of these 2 AEs was adjudicated to be related to a study drug.

**Table 3:** Additional adverse events identified by monitoring

Adverse Event	Arm	Grade	SAE	AESI	Comment
Lymphocyte count decreased	IC14	4	No	No	Lab data were automatically extracted, but no AE ECRF was completed because lymphopenia is a common AE related to severe COVID-19. AE ECRF was subsequently completed at the request of the monitor. AE judged by the investigator to be unrelated to the study drug.
Activated partial thromboplastin time prolonged	Ciproheptadine	3	No	No	No AE ECRF was completed because “elevated aPTT was an intended therapeutic effect of the heparin drip the patient was on.” AE ECRF was completed at the request of the monitor. According to the investigator, this event was “unlikely” to be related to the study drug.
Thromboembolic event	Control	3	Yes	No	The investigator checked the box for PE in the daily report form and additionally noted the occurrence in the free-text section. An additional AE ECRF was not completed at the time, but was requested during retrospective monitoring.
Lymphocyte count decreased	Control	3	No	No	Lab data were automatically extracted, but no AE ECRF was completed because lymphopenia is a common AE related to severe COVID-19. AE ECRF subsequently completed at request of monitor.
Lymphocyte count decreased	Control	3	No	No	See the grade 3 lymphocyte count above.
Anemia	Control	3	No	No	Lab data were automatically extracted but no AE ECRF was completed because anemia is a common AE related to critical illness. AE ECRF completed at request of monitor.
Seizure	Control	3	No	No	An adverse event occurred immediately after consent, prior to the administration of the study drug. The event was known to the sponsor as the patient was moved off the interventional arm. No AE ECRF was initially completed because the patient had not received the study drug. AE ECRF was subsequently completed at the request of the monitor.
GI Bleeding	Control	3	No	No	Bleeding was noted in the daily events free-text form, but no AE ECRF was completed at the time. AE ECRF subsequently completed at the request of monitor.
GI Bleeding	Cyclosporine	3	Yes	No	This AE was not noted in the daily form and no AE was completed. AE ECRF was subsequently completed at the request of monitor. AE judged by the investigator to be unrelated to the study drug.
Elevated Liver Function Tests	Narsoplimab	2	No	Yes	Lab data were automatically extracted, but no AE ECRF was completed. AE ECRF completed at request of monitor. In the opinion of the investigator, the AE was “possibly” related to the study drug.
Myocardial Infarction	Cyclosporine	1	No	No	Not recorded in the daily events report. This event was detected by a CRA query after reviewing the EHR, which resulted in a change of the daily reform and completion of AE ECRF. In the judgment of the investigator and the SWG, this event was related to COVID myocarditis, present upon admission, and was unrelated to the study drug.

Abbreviations: *AE* adverse event, *SAE* serious adverse event, *AESI* adverse event of special interest, *AE ECRF* adverse event case report form.

### Changes in protocol compliance

No new protocol deviation eCRFs were created during retrospective monitoring. There were 7 data field changes in the existing protocol deviation eCRF, none of which changed the nature of the deviation reported nor required additional reporting to the IRB.

### Cost of monitoring

The sponsor estimated expending 56,132 h by both permanent and temporary staff at a cost of $5.7 M, resulting in an estimated hourly cost of monitoring of approximately $100. According to our survey, the sites spent an estimated 4941 h by site personnel on activities related to monitoring and SDV, including 1461 h responding to queries, 1058 h onboarding monitors, and 1422 h on monitoring visits (Supplementary Table S7). Combining the efforts of the sponsor and the sites, we estimated that monitoring required 61,073 person-hours, at a total cost of $6.1 M, corresponding to $18 per data field verified and $4862 per data field error.

## Discussion

In this large multicenter platform trial, we report the evaluation of an innovative approach to clinical trial data capture, monitoring, and safety assurance without SDV that was spurred by restrictions imposed by the COVID-19 pandemic. After trial arms were closed, extensive retrospective monitoring (30% of patients), at a cost of $6.1 M, resulted in adjustments to only 0.36% of the data fields reviewed. Although these changes resulted in 9 changes to variables used in primary outcome assessment and 11 additional AE eCRFs, they had no impact on conclusions related to the efficacy, safety, or tolerability of any individual study arm, or on the number or severity of AEs attributed to study drugs. We posit that other clinical trials could use the data capture, monitoring, and safety strategy employed in I-SPY COVID to ensure high-quality data capture without the need for SDV.

The I-SPY COVID trial is by no means the first clinical trial to focus on streamlined data capture to improve data accuracy. Other COVID-19 trials, like UK RECOVERY, also relied on routinely collected health data, simple eCRFs, and no SDV^14^. However, the unique contribution of the current study is that we were able to evaluate our trial procedures due to the extensive retrospective SDV that was performed after trial arms had closed. In effect, this created a natural experiment to assess the impact of our data capture, monitoring, and safety strategy on data accuracy, trial results, and costs.

Our study illustrates the potential of digital data capture technologies to ensure high-fidelity primary data capture, instead of post hoc correction of data errors. We implemented automated transfer of electronic source data to the EDC, which has been shown to eliminate human transcription errors that are a major source of errors discovered by SDV and frees up time for study staff to focus on entering data that cannot be captured electronically^15^. In the I-SPY COVID trial, we found that electronic source data capture of laboratory results and medications was associated with a decrease in the error rate in data fields discovered during retrospective SDV. In this trial, due to limitations in the underlying EHR data structure and interoperability, only a small percentage of all data was directly captured from the EHR. We believe there is a need to expand electronic source data capture to include a greater variety and proportion of the data. We hypothesize that this could lead to error rates (and therefore the value of SDV) to decrease further^15^.

For data that cannot be captured automatically, we developed a concise daily checklist of the most important clinical events (Supplementary Fig. 1), rather than relying solely on investigator-initiated reports. We hypothesize that systematic data capture is particularly important in a trial of critically ill participants to reduce bias in reporting AEs. Traditionally, investigators report AEs on dedicated forms and judge whether any AE could be attributable to a study drug. However, critically ill patients experience a high volume of AEs, the majority of which are related to the underlying disease, making it difficult for investigators to discern a safety signal from noise, and creating the potential for reporting bias^1^. It is possible— although not yet proven—that the checklist, paired with daily extraction of laboratory data, creates a more systematic and less biased strategy to collect AEs and could allow for more rigorous causal inference.

Another important strategy we employed was to strengthen centralized safety monitoring using the SWG. The SWG was a novel safety oversight body that replaced and expanded on the role of a traditional medical monitor. Unlike a medical monitor, the SWG was actively involved in the management of study drugs and emerging toxicities, as an advisory group to the DMC. As an illustration, an arm focusing on the drug razuprotafib was terminated after the SWG evaluated the safety run-in and concluded that although the drug was likely safe, the level of additional monitoring required for this particular agent would not be a good use of resources during the early pandemic. Unlike an individual investigator or a medical monitor, the SWG was able to adjudicate events to ensure the DMC could properly review the number of AEs and the distribution of laboratory values for each agent in aggregate, enabling early detection of toxicity signals and data irregularities^16,17^. Because the SWG relied on the experience of the lead investigators for each drug, the total cost was modest at less than $50,000 per year in salary support, far below the cost of retrospective monitoring.

Retrospective SDV confirmed the fidelity of our safety strategy. Although retrospective monitors requested sites to enter 11 additional AE eCRFs, our systematic data collection had already recorded 9 of these AEs in different eCRFs, as part of the daily checklist and daily labs. We argue that asking investigators to complete AE eCRFs for events that are common in critical illness due to COVID-19, are unlikely to be related to the study drugs, and that have already been captured in other eCRFs, is not a prudent use of resources. The two additional AEs that had not previously been captured were approximately 1% of the total number of AEs recorded amongst participants that underwent monitoring and did not change our conclusions regarding the safety profile of study drugs.

These results reinforce the idea that traditional SDV has limited utility at an exceedingly high cost. Previous studies have shown that even after complete SDV, no form of monitoring can ensure data that is 100% accurate^18–20^, and that SDV is unlikely to impact the main conclusions of a trial^21^. In fact, allocating resources to verifying low-value data elements can divert attention from critical data, which can impair the efficiency and safety of a trial^22–26^. Similar to prior studies, we found that there is variation in the error rate across sites and eCRFs. Therefore, trials should be designed to ensure that data are free from non-random, systematic errors that matter^27^, in accordance with “quality by design”. This concept has led to risk-based monitoring frameworks that focus on SDV of only high-risk data elements and sites that may be performing poorly^28–30^. There is substantial evidence that risk-based monitoring with only partial SDV involving less than 50% of data elements is effective at safeguarding trial integrity^20,31^. Our results add to this evidence by demonstrating that a strategy focused on capturing data accurately and systematically in the first place, with targeted safety monitoring but 0% SDV, can ensure accurate trial results.

We spent in excess of 60,000 h on monitoring, for 333 patients and 10,101 eCRFs—accounting for fewer than a third of eCRFs. If monitoring and SDV had been performed for all patients, the total effort and cost could have exceeded $18 M and 180,000 h. While some of this time was spent on travel, meeting with sites, managing queries to sites, and compiling reports and presentations, this extraordinary time requirement is also a function of the volume of data required for modern clinical trials and the difficulty of retrospectively reviewing EHR records to verify individual data fields. There has been a substantial increase in the complexity of trial data collection, with eCRFs that can now run to hundreds of pages^32,33^. In comparison, the GUSTO trial in the early 1990s that compared different thrombolytic strategies in acute myocardial infarction, used a simple, three-page eCRF, despite the high-risk nature of the intervention and trial population^33^. We hope that electronic source data capture and streamlined data capture strategies like those employed in the I-SPY COVID trial could help reduce the complexity of clinical trial data capture.

Based on the experience in I-SPY COVID, we have taken these lessons forward into improving and innovating data collection in our I-SPY oncology platform trials. In the I-SPY2.2 neoadjuvant platform trial for molecularly high-risk early breast cancer, we are focusing on standardizing data collection on immune-related AEs (irAEs) and AEs of special interest (AESIs). We are implementing a checklist of AESI and irAEs that is completed at every study visit, which will speed up data entry, and give the SWG access to complete information in real time, allowing them to identify new emerging AEs rapidly to address and potentially prevent them before the study is complete.

We are also working to use streamlined data collection tools to bridge the divide between clinical care and clinical research. A major barrier towards achieving this vision remains that clinical trials mainly rely on separate data infrastructure and data collection tools from clinical care^34^. This separation makes it difficult to use routinely generated health data to power continuous improvement in healthcare^35^. One goal of I-SPY COVID was to design data entry systems of sufficient simplicity that clinicians could complete them during routine care. The checklist was designed to capture information essential for clinicians caring for patients with critical illness. Similarly, in I-SPY2.2, we are working to optimize the clinic workflow and data entry forms to routinely capture data that is needed for both care and research in a structured format, instead of relying on unstructured notes for clinical care and duplicative data entry for research. This data could then be used to pre-fill eCRFs for clinical trials, but also for clinical care, for example, by creating visualizations that help clinicians access critical information more efficiently, or by pre-filling clinical notes. However, operational and organizational challenges remain to be addressed. For example, during I-SPY COVID, hospital leadership prevented us from pre-filling clinical notes with data entered on the daily eCRF, including the checklist, due to concerns about the validity of the resulting notes for billing.

There are several limitations to this study. The most important thing is that retrospective monitoring was completed over a 7-month period, during which other trial activities, such as meetings of the DMC, SWG, and data entry and cleaning from sites, remained ongoing. While we performed a detailed audit to determine the reason for any changes in the primary outcome and AE reports, it is possible that the fact that monitoring was ongoing changed the behavior of the sites, the SWG, or the DMC, so that these groups performed better or worse than they would have in the absence of monitoring. In addition, given the large number of changes in the EDC system, it was not possible to determine the etiology of data changes not involving AEs or the primary outcome. Therefore, the data changes reported here represent the totality of changes during the time period when retrospective SDV was conducted, which could overestimate the impact of retrospective SDV. In addition, the estimate of SDV costs relied in part on a survey completed by the sites, which could be affected by recall bias, and also used a single estimate of the hourly cost of monitoring based on the sponsor’s experience, while the true cost per hour may have varied across the sites. Finally, our results should be viewed in the context of the COVID-19 pandemic and the specific processes designed for this trial, so the findings may not necessarily generalize to all trials. However, it is worth noting that this was a platform trial that included 11 active arms and 42 sites, including both academic and non-academic medical centers, indicating that the processes developed for this trial can be implemented across a wide and diverse range of sites.

In conclusion, we developed a streamlined approach to data collection and monitoring for the I-SPY COVID trial focused on mission-critical data elements to reduce the burden of data collection. The accuracy of this approach was confirmed by retrospective monitoring with extensive SDV at a cost of $6 M, which resulted in changes to 0.36% of the raw data fields but did not identify any systematic errors or affect any conclusions of the trial. This confirms that traditional SDV is labor intensive, expensive, and detracts from focusing on other mission critical trial related activities and can potentially be eliminated by incorporating systematic data capture through checklists, electronic source data capture, and centralized monitoring.

## Supplementary information

Supplementary Information

description of additional supplementary files

Supplementary Data 1

reporting summary

## Data Availability

The data reported in this paper are maintained by the study sponsor, Quantum Leap Healthcare Collaborative (QLHC). Data can be shared with investigators external to the trial following approval by QLHC and the I-SPY COVID Data Access and Publications Committee, beginning at the time of publication of this article. This request can be initiated by contacting the corresponding author of this manuscript. The data underlying Fig. 2A (raw counts of changes made for each type of eCRF) can be found in Supplementary Data 1. Raw counts for Fig. 2B are reported in the figure itself.

## References

[1] May, M. Clinical trial costs go under the microscope. *Nat. Med*. 10.1038/d41591-019-00008-7 (2019).

[2] Getz, K. A. & Campo, R. A. Trends in clinical trial design complexity. *Nat. Rev. Drug Discov.* **16**, 307–307 (2017). 28417986 10.1038/nrd.2017.65

[3] Sertkaya, A., Birkenbach, A., Berlind, A. & Eyraud, J. *Examination of Clinical Trial Costs and Barriers for Drug Development*. https://aspe.hhs.gov/reports/examination-clinical-trial-costs-barriers-drug-development-0 (2014).

[4] Love, S. B. et al. What is the purpose of clinical trial monitoring?. *Trials* **23**, 836 (2022). 36183080 10.1186/s13063-022-06763-2 PMC9526458

[5] Institute of Medicine (US) Forum on Drug Discovery, Development and Translation. *Transforming Clinical Research in the United States*. (National Academies Press (US), Washington, DC, 2010). 10.17226/12900. 21210556

[6] Stansbury, N. et al. Risk-based monitoring in clinical trials: increased adoption throughout 2020. *Ther. Innov. Regul. Sci.* **56**, 415–422 (2022). 35235192 10.1007/s43441-022-00387-z PMC8889871

[7] Files, D. C. et al. I-SPY COVID adaptive platform trial for COVID-19 acute respiratory failure: rationale, design and operations. *BMJ Open* **12**, e060664 (2022). 35667714 10.1136/bmjopen-2021-060664 PMC9170797

[8] Park, J. W. et al. Adaptive randomization of neratinib in early breast cancer. *N. Engl. J. Med*. **375**, 11–22 (2016). 27406346 10.1056/NEJMoa1513750 PMC5259558

[9] Rugo, H. S. et al. Adaptive randomization of veliparib–carboplatin treatment in breast cancer. *N. Engl. J. Med.* **375**, 23–34 (2016). 27406347 10.1056/NEJMoa1513749 PMC5259561

[10] World Health Organization. WHO R&D Blueprint: Novel Coronavirus, COVID-19 Therapeutic Trial Synopsis. https://www.who.int/blueprint/priority-diseases/key-action/COVID-19_Treatment_Trial_Design_Master_Protocol_synopsis_Final_18022020.pdf (2020).

[11] Food and Drug Administration. Source Data Capture from Electronic Health Records (EHRs): Using Standardized Clinical Research Data (OneSource Phase I). *FDA*. https://www.fda.gov/science-research/advancing-regulatory-science/source-data-capture-electronic-health-records-ehrs-using-standardized-clinical-research-data (2021).

[12] Office of the National Coordinator for Health IT. Standardized API for patient and population services. https://www.healthit.gov/test-method/standardized-api-patient-and-population-services (2025).

[13] The I-SPY COVID Consortium et al. Report of the first seven agents in the I-SPY COVID trial: a phase 2, open label, adaptive platform randomised controlled trial. *eClinicalMedicine* **58**, 101889 (2023). 36883141 10.1016/j.eclinm.2023.101889 PMC9981330

[14] RECOVERY Collaborative Group et al. Dexamethasone in Hospitalized Patients with Covid-19. *N. Engl. J. Med.* **384**, 693–704 (2020). 32678530 10.1056/NEJMoa2021436 PMC7383595

[15] Nordo, A. H. et al. A comparative effectiveness study of eSource used for data capture for a clinical research registry. *Int. J. Méd. Inform.* **103**, 89–94 (2017). 28551007 10.1016/j.ijmedinf.2017.04.015 PMC5942198

[16] Bakobaki, J. M. et al. The potential for central monitoring techniques to replace on-site monitoring: findings from an international multi-centre clinical trial. *Clin. Trials* **9**, 257–264 (2012). 22064687 10.1177/1740774511427325

[17] Lindblad, A. S. et al. Central site monitoring: results from a test of accuracy in identifying trials and sites failing Food and Drug Administration inspection. *Clin. Trials* **11**, 205–217 (2014). 24296321 10.1177/1740774513508028

[18] Smith, C. T. et al. The value of source data verification in a Cancer Clinical Trial. *PLoS ONE* **7**, e51623 (2012). 23251597 10.1371/journal.pone.0051623 PMC3520949

[19] Andersen, J. R. et al. The impact of source data verification on data quality in clinical trials. *Br. J. Clin. Pharmacol.* **79**, 660–668 (2015). 25327707 10.1111/bcp.12531 PMC4386950

[20] Fougerou-Leurent, C. et al. Impact of a targeted monitoring on data-quality and data-management workload of randomized controlled trials: a prospective comparative study. *Br. J. Clin. Pharmacol.* **85**, 2784–2792 (2019). 31471967 10.1111/bcp.14108 PMC6955406

[21] Embleton-Thirsk, A. et al. Impact of retrospective data verification to prepare the ICON6 trial for use in a marketing authorization application. *Clin. Trials* **16**, 502–511 (2019). 31347385 10.1177/1740774519862528 PMC6801797

[22] Sargent, D. J. & George, S. L. Clinical Trials Data Collection: when less is more. *J. Clin. Oncol.* **28**, 5019–5021 (2010). 20921455 10.1200/JCO.2010.31.7024

[23] Kaiser, L. D. et al. Optimizing collection of adverse event data in Cancer Clinical Trials supporting supplemental indications. *J. Clin. Oncol.* **28**, 5046–5053 (2010). 20921453 10.1200/JCO.2010.29.6608 PMC3018355

[24] Landray, M. J. et al. Improving public health by improving clinical trial guidelines and their application. *Eur. Hear. J.* **38**, ehx086 (2017). 10.1093/eurheartj/ehx086 PMC5837481 28329235

[25] Wieten, S., Burgart, A. & Cho, M. Resource allocation in COVID-19 Research: which trials? Which patients?. *Am. J. Bioeth.* **20**, 86–88 (2020). 32716767 10.1080/15265161.2020.1779392

[26] Morin, D. J. Harmonizing protocol complexity with resource management and capacity planning at clinical research sites. *Ther. Innov. Regul. Sci.* **54**, 978–987 (2020). 31933181 10.1007/s43441-020-00120-8

[27] Manasco, P. & Bhatt, D. L. Evaluating the evaluators—developing evidence of quality oversight effectiveness for clinical trial monitoring: Source data verification, source data review, statistical monitoring, key risk indicators, and direct measure of high risk errors. *Contemp. Clin. Trials* **117**, 106764 (2022). 35436623 10.1016/j.cct.2022.106764

[28] Sprenger, K., Nickerson, D., Meeker-O’Connell, A. & Morrison, B. W. Quality by Design in Clinical Trials. *Ther. Innov. Regul. Sci.* **47**, 161–166 (2012). 10.1177/0092861512458909 30227529

[29] Meeker-O’Connell, A. et al. Enhancing clinical evidence by proactively building quality into clinical trials. *Clin. Trials*, **13**, 439–444 (2016). 27098014 10.1177/1740774516643491 PMC4952025

[30] Fielman, K. T., Soto-Ruiz, K. M. & Manasco, P. K. Oversight method identifies critical errors missed by traditional monitoring approaches. *Appl. Clin. Trials*, https://www.appliedclinicaltrialsonline.com/view/oversight-method-identifies-critical-errors-missed-by-traditional-monitoring-approaches (2023).

[31] Brosteanu, O. et al. Risk-adapted monitoring is not inferior to extensive on-site monitoring: results of the ADAMON cluster-randomised study. *Clin. Trials* **14**, 584–596 (2017). 28786330 10.1177/1740774517724165 PMC5718334

[32] Kramer, J. M., Smith, P. B. & Califf, R. M. Impediments to Clinical Research in the United States. *Clin. Pharmacol. Ther.* **91**, 535–541 (2012). 22318614 10.1038/clpt.2011.341

[33] The GUSTO Investigators. An International Randomized Trial comparing four thrombolytic strategies for acute myocardial infarction. *N. Engl. J. Med.* **329**, 673–682 (1993). 8204123 10.1056/NEJM199309023291001

[34] Angus, D. C. et al. The Integration of Clinical Trials with the practice of medicine. *J. Am. Med. Assoc.* **332**, 153–162 (2024). 10.1001/jama.2024.4088 PMC12045079 38829654

[35] Institute of Medicine. *The Learning Healthcare System*. (National Academies Press (US), Washington, DC, 2007). 10.17226/11903.

