# Author Correction: Systematic data capture reduces the need for source data verification: exploratory analysis from a phase 2 multicenter randomized controlled platform trial

**DOI:** 10.1038/s43856-026-01414-y

**Published:** 2026-03-24

**Authors:** Ali B. Abbasi, Kathleen D. Liu, Derek W. Russell, D. Clark Files, Karl W. Thomas, Fady Youssef, Sheetal Gandotra, Andrea Discacciati, Noha Lim, Adam L. Asare, Martin Eklund, Michael Matthay, Laura J. Esserman, I-SPY COVID Investigators, Neil R. Aggarwal, Ellen L. Burnham, Carrie Higgins, Jeff McKeehan, Timothy Albertson, Angela Haczku, Erin Hardy, Richart Harper, Brian Morrissey, Christian Sandrock, Sara Auld, Philip Yang, Joshua Detelich, Gavin Harris, Katherine Nugent, Max Adelman, Jeremy R. Beitler, Anita Darmanian, Amy L. Dzierba, Ivan Garcia, Katarzyna Gosek, Purnema Madahar, Aaron M. Mittel, Justin Muir, Amanda Rosen, John Schicchi, Alexis L. Serra, Romina Wahab, Paul Berger, Carolyn S. Calfee, Melissa Coleman, Alejandra Jauregui, Nathan Cobb, Rajiv Sonti, Amen M. Hamed, Alessio Crippa, Andrea Discacciati, Martin Eklund, Laura Esserman, D. Clark Files, Karl Thomas, Kevin W. Gibbs, Leigha Landreth, Mary LaRose, Lisa Parks, Adina Wynn, Eliot Friedman, Derek W. Russell, Donna Harris, Abhishek Methukupally, Siddharth Patel, Sheetal Gandotra, Kashif Khan, Se Fum Wong, Albert Yen, Jonathan Koff, Lindsie Boerger, John Kazianis, Santhi Kumar, Kathleen D. Liu, Thomas R. Martin, Mark M. Wurfel, Michael A. Matthay, Brian Daniel, Nuala J. Meyer, Caroline A. G. Ittner, Nilam S. Mangalmurti, John P. Reilly, Timothy Obermiller, Philip A. Robinson, Farjad Sarafian, Usman Shah, Richard G. Wunderink, G. R. Scott Budinger, Helen K. Donnelly, Benjamin D. Singer, Fady A. Youssef, Daniel Belvins, Catherine Nguyen, Alexis Suarez, Maged A. Tanios, Scott Fields, James Hurst-Hopf, Lamorna Brown Swigart, Christina Creel-Bulos, Christina Spainhour, Ari Moskowitz, Praveen Vijhani, Adrienne M. Casciato, Vaney Capetillo, Kenenth Wei, Tracie Huynh, Anna Rodriguez-Vasquez, Joseph L. Nates, Jessica Suarez, Siddharth Nair, Sandya Samavedam, Michael Bernstein, Christopher Jordan, Daniel H. Kett, Karla Leon Escalona, Richard A. Lee, Kenneth Remy, Ali Zarrinpar, Robert Hyzy, Kristine Nelson, Caroline Quill, Emilio Mazza, Kristin Broderick, Jermiah Hayanga, Ana Costa Monteiro, Joseph Levitt, Ruixiao Lu, Paul Henderson, Adam Asare, Imogene Dunn, Alejandro Botello Barragan

**Affiliations:** 1 University of California San Francisco, San Francisco, CA USA; 2 University of Alabama at Birmingham, Birmingham, AL USA; 3 Wake Forest University School of Medicine, Winston-Salem, NC USA; 4 MemorialCare Long Beach Medical Center, Long Beach, CA USA; 5 Karolinska Instituet, Stockholm, Sweden; 6 Quantum Leap Healthcare Collaborative, San Francisco, CA USA; 7 Pulmonary Sciences & Critical Care, University of Colorado, Aurora, CO USA; 8 Division of Pulmonary, Critical Care and Sleep Medicine, University of California Davis, Sacramento, CA USA; 9 Department of Medicine, Emory University, Atlanta, GA USA; 10 Centre for Acute Respiratory Failure, Columbia University, New York, NY USA; 11 Department of Critical Care, Sanford Health, Sioux Falls, SD USA; 12 Division of Pulmonary, Critical Care, Allergy and Sleep Medicine, University of California San Francisco, San Francisco, CA USA; 13 Pulmonary and Critical Care Medicine, Georgetown University, Washington, DC USA; 14 Department of Medical Epidemiology and Biostatistics, Karolinska Institutet, Solna, Sweden; 15 Department of Surgery, University of California San Francisco, San Francisco, CA USA; 16 Pulmonary and Critical Care Medicine, Wake Forest University, Winston-Salem, NC USA; 17 Department of Medicine, Main Line Health, Wynnewood, PA USA; 18 Pulmonary, Allergy, and Critical Care Medicine, University of Alabama Birmingham, Birmingham, AL USA; 19 Pulmonary Critical Care, University of Maryland Medical Center, Baltimore, MD USA; 20 Critical Care Medicine, Keck School of Medicine University of Southern California, Los Angeles, CA USA; 21 Pulmonary, Critical Care, and Sleep Medicine, Yale University, New Haven, CT USA; 22 Division of Pulmonary, Critical Care and Sleep Medicine, Keck School of Medicine University of Southern California, Los Angeles, CA USA; 23 Divisions of Nephrology and Critical Care Medicine, University of California San Francisco, San Francisco, CA USA; 24 Pulmonary, Critical Care and Sleep Medicine, University of Washington, Seattle, WA USA; 25 Critical Care Medicine, University of California San Francisco, San Francisco, CA USA; 26 Department of Medicine, University of Pennsylvania Perelman School of Medicine, Philadelphia, PA USA; 27 Critical Care Medicine, Logan Health Research Institute, Kalispell, MT USA; 28 Infectious Disease, Hoag Medical Group, Newport Beach, CA USA; 29 Department of Medicine, Pulmonary and Critical Care Division, Northwestern University Feinberg School of Medicine, Chicago, IL USA; 30 Department of Internal Medicine, Long Beach Memorial Medical Centre, Long Beach, CA USA; 31 Investigational Drug Service, University of California San Francisco, San Francisco, CA USA; 32 Department of Laboratory Medicine, University of California San Francisco, San Francisco, CA USA; 33 Department of Anesthesiology, Emory University, Atlanta, GA USA; 34 Department of Emergency Medicine, Emory University, Atlanta, GA USA; 35 Division of Critical Care Medicine, Montefiore Medical Centre, Bronx, NY USA; 36 Pulmonary and Critical Care Medicine, DHR Health, Edinburg, TX USA; 37 Kaiser Permanente Los Angeles Medical Center, Los Angeles, CA USA; 38 Department of Critical Care Medicine, Division of Anesthesiology, Critical Care Medicine and Pain Medicine, MD Anderson Cancer Center, Houston, TX USA; 39 Critical Care Medicine, Mercy Hospital, Springfield, MO USA; 40 Critical Care Medicine, Stamford Health, Stamford, CT USA; 41 Critical Care Medicine, University of Miami, Miami, FL USA; 42 Pulmonary and Critical Care Medicine, University of California Irvine, Irvine, CA USA; 43 Critical Care Medicine, Innovations in Pulmonology, Critical Care & Sleep Medicine, Cleveland, OH USA; 44 Division of Transplantation and Hepatobiliary Surgery, University of Florida, Gainesville, FL USA; 45 Critical Care Medicine, University of Michigan, Ann Arbor, MI USA; 46 Division of Pulmonary & Critical Care Medicine, University of Rochester, Rochester, MN USA; 47 Pulmonary, Critical Care and Sleep Medicine, Virtua Our Lady of Lourdes Hospital, Evesham, NJ USA; 48 Surgical Critical Care, West Virginia University, Morgantown, WV USA; 49 Pulmonary and Critical Care, University of California Los Angeles, Los Angeles, CA USA; 50 Pulmonary, Allergy and Critical Care Medicine, Stanford University, Stanford, CA USA

**Keywords:** Clinical trials, Drug development

Correction to: *Communications Medicine* 10.1038/s43856-025-01126-9, published online 29 October 2025

In this article the author name Fady Youssef was incorrectly written as Fady Yousef. The original article has been corrected.

