# Supplementary material for: Systematic data capture reduces the need for source data verification: exploratory analysis from a phase 2 multicenter randomized controlled platform trial
Source: Commun Med (Lond). 2025 Oct 29;5:444. doi: 10.1038/s43856-025-01126-9 (PMC12572212; doi:10.1038/s43856-025-01126-9)
Supplement: Supplementary file 1 — Supplementary Information [file 43856_2025_1126_MOESM1_ESM.pdf]

SUPPLEMENTARY INFORMATION

Innovative approaches to systematic data capture can reduce the need for source data verification: Results from I-SPY COVID, a large phase II multicenter randomized controlled platform trial

Ali B. Abbasi MD,1 Kathleen D. Liu MD, PhD,1 Derek W. Russell MD,2 D. Clark Files MD,3 Karl W. Thomas MD,3 Fady Yousef MD,4 Sheetal Gandotra MD2, Andrea Discacciati, PhD5, Adam L. Asare PhD,1,6 Martin Eklund PhD,5 Michael Matthay MD,1 Laura J. Esserman MD MBA,1 I-SPY COVID Investigators

TABLE OF CONTENTS

1. Supplementary Methods 3
1.1 Estimation of hours required for monitoring by the sites.......... 3
1.2 Arm termination reports pre- and post-monitoring 3
2. Supplementary Results 3
2.1 Changes in arm termination reports pre- and post-monitoring 3
3. Supplementary Figures 4
Supplementary Figure 1: I-SPY COVID Daily Data eCRF.......... 4
Supplementary Figure 2: Automated laboratory event grading 5
Supplementary Figure 3: Process for adjudicating changes to the primary outcome 6
Supplementary Figure 4: Percent of data fields changed by CRF and by site 7
Supplementary Figure 5: Median posterior hazard ratios and 95% CI of arms before/after monitoring 8
3. Supplementary Tables 9
Supplementary Table 1: Electronic Case Report Forms utilized in the I-SPY COVID trial classified by risk level 9
Supplementary Table 2: List of question on survey distributed to sites to estimate the cost of retrospective monitoring to sites. 10
Supplementary Table 3: Data changes in the EDC by eCRF type between 6/1/2022-12/31/2022. 11
Supplementary Table 4: Distribution of patients enrolled across clinical sites 12
Supplementary Table 5: Data changes in the EDC during the period of retrospective monitoring between 6/1/2022-12/31/2022 13
Supplementary Table 6: Additional AE ECRFs completed during 6/1/2022-12/31/2022 by source and grade 13
Supplementary Table 7: Calculation of hours used for monitoring by the sites.......... 14

1. Supplementary Methods

1.1 Estimation of hours required for monitoring by the sites.

To estimate the total number of hours required for monitoring by the sites, we conducted a survey of the sites, where we asked each site to estimate the number of hours required for the following tasks: onboarding monitors, remote monitoring visits, in-person monitoring visits, time to respond to each type of query (daily form, adverse events, eligibility and randomization, study drug administration, ePRO, lab results, protocol deviation, termination). There were several rare query types that were not represented in the survey, so we used the average time estimate across all queries in the survey. To obtain an estimate of the number of queries related to retrospective monitoring and SDV, we used the number of queries during the period 6/1/2022-12/31/2022 and corrected for the "background" volume of queries in the trial by subtracting the average number of queries per month that occurred during all other periods of the trial. Finally, we multiplied the average estimate of the number of hours per query with the number of queries that occurred during the period of retrospective monitoring. Details of this calculation are in Table S7.

1.2 Arm termination reports pre- and post-monitoring

To understand whether the changes we identified could have had any impact on trial conclusions, we compared arm termination reports generated before and after the completion of retrospective monitoring. The details of the trial data analysis are specified in the trial protocol. Briefly, for each agent, the pre-specified intention-to-treat analyses were performed for the co-primary outcomes of all-cause death and time to recovery. Futility was assessed based on the posterior distributions of the hazard ratios(HR) for recovery and all-cause death (adjusted for baseline COVID-19 level). It is important to note that, in addition to retrospective monitoring, there were additional changes in the Electronic Data Capture system (EDC) due to activities other than monitoring, from ongoing work of the SWG (reviewing all deaths, and reported AEs that were possibly, probably or definitely related to drug), DMC, data cleaning and entry from the sites, and changes to the statistical analysis plan that occurred during the same time period. Due to the complexity of the EDC in this large trial, it is impossible to establish an etiology of each change, and our results represent the *en-bloc* impact of all changes that occurred during this time period. Therefore, the results of this analysis are likely to overestimate the degree of change in conclusions that resulted from monitoring.

2. Supplementary Results

2.1 Changes in arm termination reports pre- and post-monitoring

When comparing analysis of the EDC pre- and post-monitoring, there were no changes in the conclusions of any of the 11 study arms. Specifically, there were only minimal changes in the median posterior hazard ratios for recovery or death (Supplementary Figure 5). Due to a small change in the SAP pre- and post-monitoring, the most common change was related to the number of participants included in the concurrent control group. The number of participants in each arm's respective control group changed by an average of 4.5 participants, and the size of interventional arm groups changed by an average of 0.7 participants. These changes resulted in small changes in the summary statistics. Due to these changes in the raw data, the median posterior HR for death and recovery and their 95% credible intervals also changed slightly (Supplementary Figure 5)

3. Supplementary Figures

Supplementary Figure 1: I-SPY COVID Daily Data eCRF

The goal was that the clinical events checklist could be completed by the ICU team on rounds, and the labs would be automatically pulled using the OneSource electronic source capture tool to reduce error rates and effort. We estimate that after implementation of OneSource, data entry on the daily data eCRF could be completed in about 2-3 minutes.

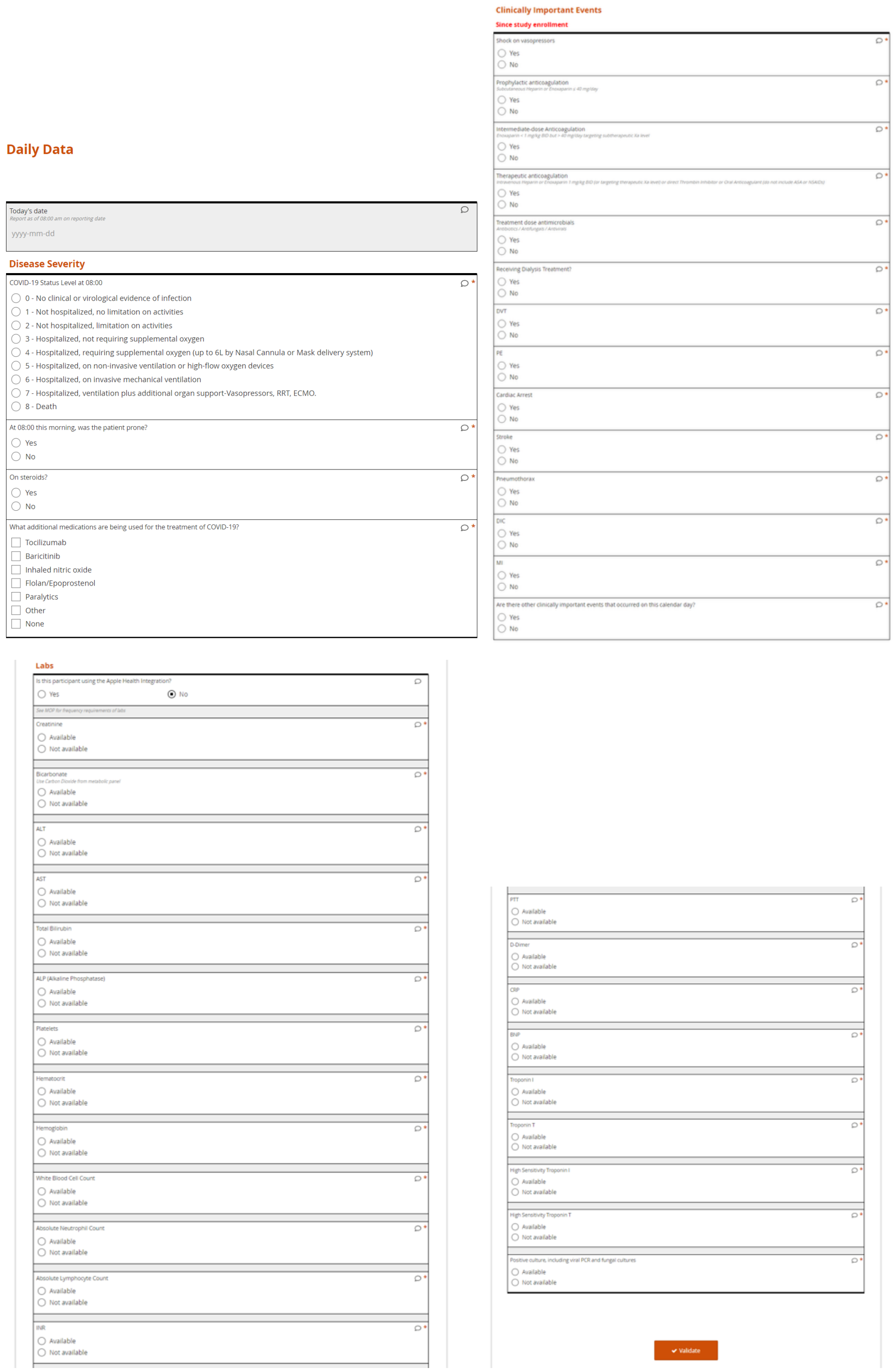

Supplementary Figure 2: Automated laboratory event grading

Example of the automated laboratory event grading and visualization provided to the data monitoring committee, showing the occurrence of frequency of laboratory adverse events by study arm, compared to the control group.

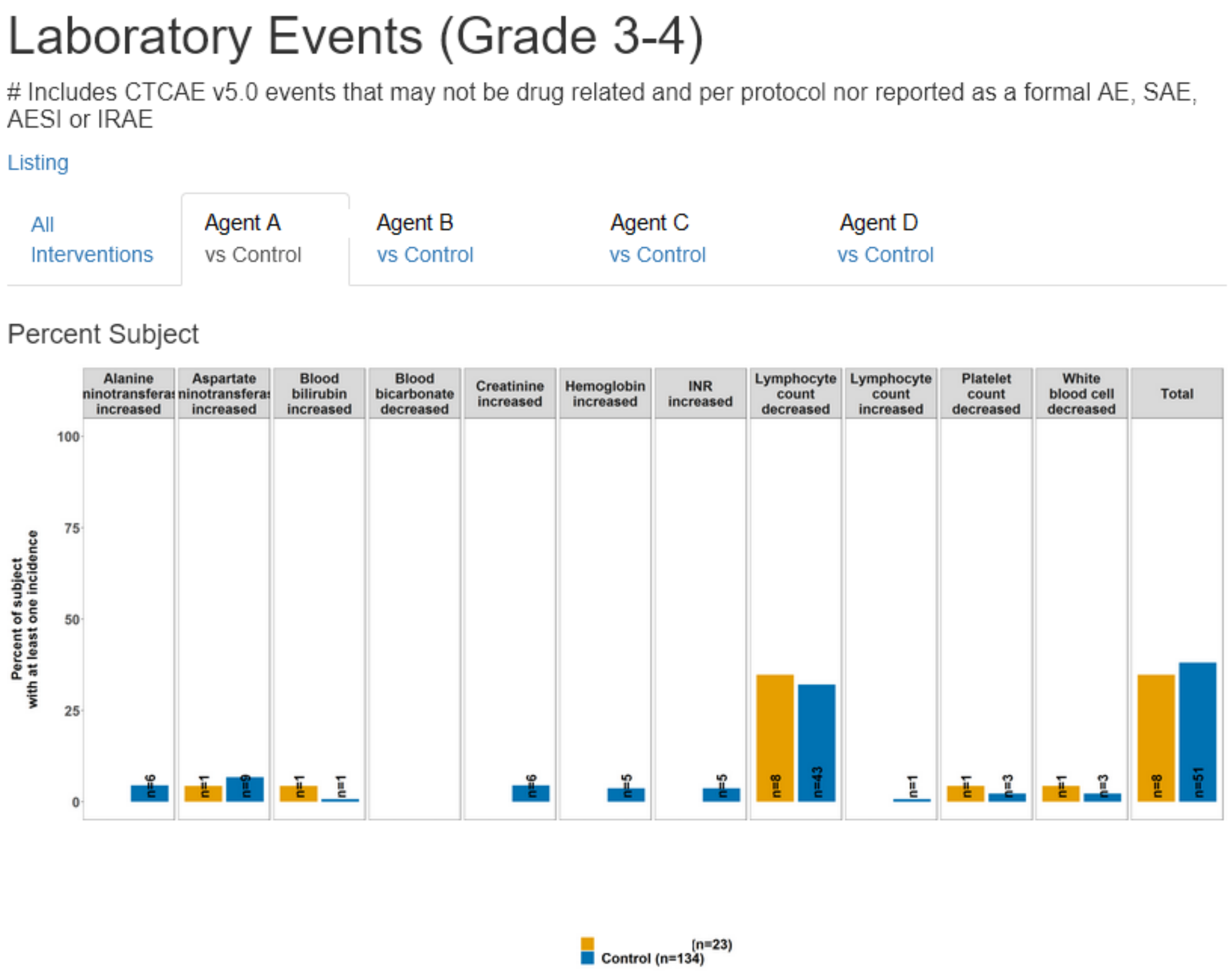

Supplementary Figure 3: Process for adjudicating changes to the primary outcome

This review was undertaken for each change in the primary outcome type and day of occurrence that occurred between 6/1/2022-12/31/2022 to determine whether the change could have been related to retrospective monitoring, or was initiated by the sites, or a centralized monitoring body.

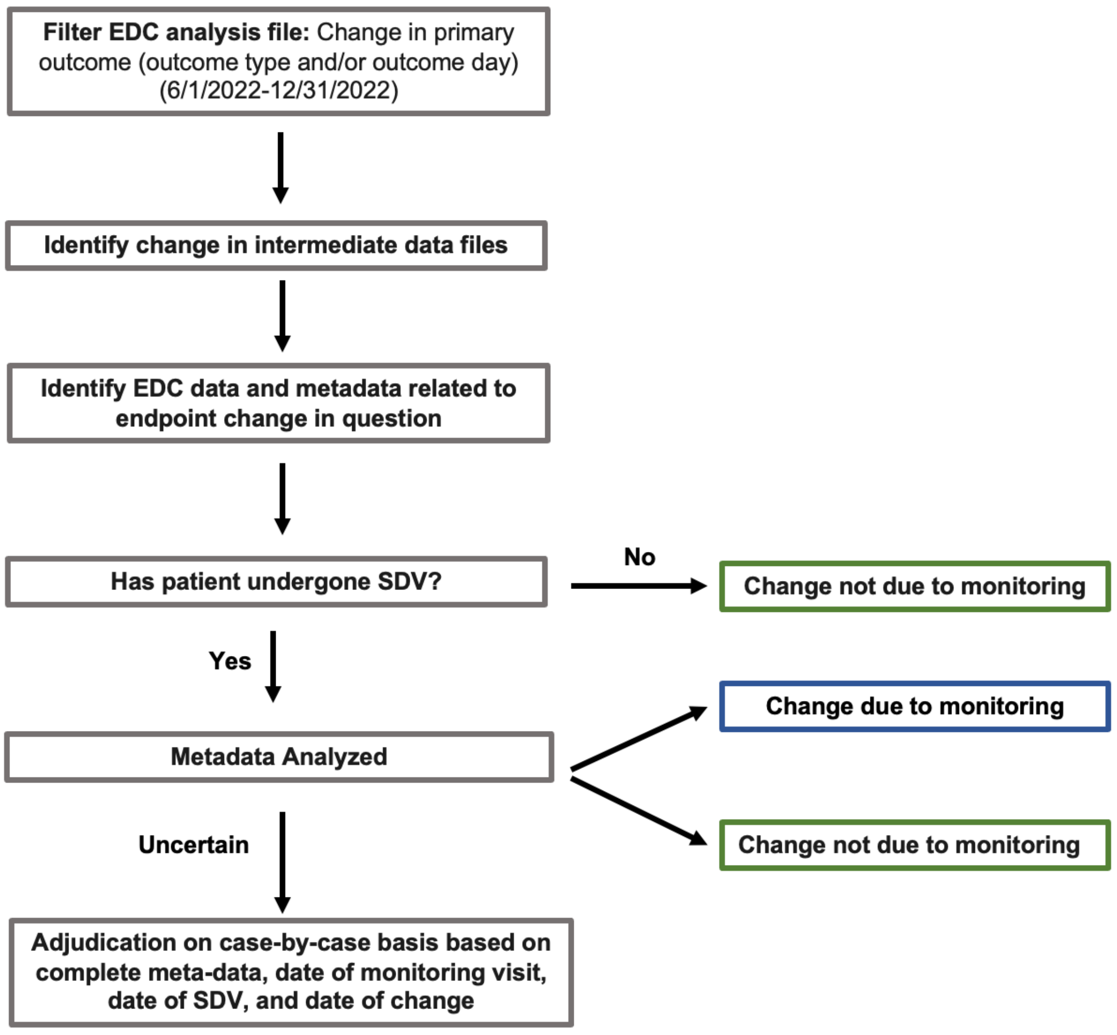

Supplementary Figure 4: Percent of data fields changed by CRF and by site

CRF ID (columns) by Site ID (rows)

Site ID	1	2	3	4	5	6	7	8	9	10	11	12	13	14	15	16	17	18	19	20	21	22	23	24	25	All
1		0.0	0.0	0.0	0.0			0.0		0.0	0.0		0.0	0.0	0.0	0.0	0.0			0.0						0.0
2	0.0	0.0	0.0	0.0				0.0		0.3	0.0	0.0	0.0	0.0	0.0	0.0										0.0
3	6.7	0.0	0.0	0.0	0.0	0.4	0.7	0.0		0.5	0.0	0.0	0.0	0.0	0.0	0.0	0.0	0.0								0.0
4	3.8	0.0	0.0	0.5		0.2	0.0	1.2	0.0	0.0	0.0		0.0	0.0	0.0				0.0							0.1
5		0.2	0.0	0.0	0.0	1.0		7.7	0.0	0.1	0.3	0.0	0.0	0.0	0.0	0.0	0.0		0.0				0.0	0.0	0.0	0.1
6	0.0	1.4	0.0	0.2		0.4	0.0	0.0		0.1	0.0		0.0	0.0	0.0		0.0									0.1
7		0.4	0.0	0.5	0.0	1.6		0.0		0.1	0.5	0.0	0.0	0.0	0.0											0.1
8		0.0	0.0	0.0		0.8		0.0		0.1	0.0	0.0	0.0	0.0	0.0	0.0										0.2
9		0.0	0.0	4.0						0.1	0.0	0.0		0.0	0.0	0.0	0.0									0.2
10		0.0		0.0				0.0		0.0	0.0	12.5		0.0	0.0	0.0				0.0						0.2
11		1.3	0.0	8.6			0.0	0.0		0.3	3.0	0.0	0.0	0.0	0.0	0.0										0.2
12		0.4	6.3	0.0	0.0	0.0	0.0			0.2	0.0	0.0	0.0	0.0	0.0											0.2
13	2.4	0.1	1.1	3.0	0.0	0.5	1.1	0.4		0.1	0.0	0.0	0.0	0.0	0.0	0.0		0.0			0.0	0.0				0.2
14		0.0	0.0	0.0						0.0	0.0	0.0	0.0	0.0	0.0	0.0										0.3
15		1.8	7.1	1.0		1.4	0.0	0.0		0.2	0.0	0.0	0.0	0.0	0.2	0.0										0.3
16		1.1	0.0	1.1		0.2			0.0	0.6	0.0	0.0	0.0	0.0	0.0	0.0										0.4
17	0.0	0.8	0.0	0.0		0.4	0.0	0.7		0.2	0.3	0.0	0.0	0.7	0.0				0.0							0.4
18	14.3	0.0	0.0	0.0	0.0					0.0	0.0	0.0		0.0	0.0	0.0				0.0						0.4
19	0.0	0.0	0.0	0.0		1.7			0.0	0.2	0.0	0.0		0.0	0.0											0.4
20	0.0	0.9	0.0	0.0	0.0	0.0	0.0			0.5	1.6	0.0	0.0	2.1	0.0	0.0				0.0						0.4
21	0.0	0.6	0.0	2.8	25.0	0.0		0.0	0.0	0.6	0.0	0.0	0.0	0.0	0.0	0.0			0.0							0.5
22	5.4	2.2	2.2	0.4	0.0	1.4	0.7	3.7		0.5	0.0	0.7	0.0	0.0	0.0	0.0	0.0	0.0								0.5
23		0.8	0.0	0.4		0.3	0.3			0.8	1.9	0.0	5.9	0.0	0.0	0.0										0.5
24		4.3	0.0	0.0		1.3				0.6	0.0	0.0	0.0	0.0		0.0										0.7
25	0.0	7.3	3.6	1.4	0.0	0.1	1.7		0.7	0.8	0.5	2.0	0.0	0.0	0.0	0.0		0.0								0.8
26	0.0	1.9	0.0	0.1		0.6	0.0	0.0		0.3	0.3	0.4	0.0	0.0	0.0	0.0	0.0	0.0					0.0	0.0		0.8
27		6.8	0.0	0.0		0.4		0.0	0.0	1.1	1.9	1.8	0.0	0.0	0.0	0.0										0.9
28		2.5	6.3	1.3		1.6	3.2	0.0	0.0	1.1	0.3	0.0	0.0	0.0	0.0	0.0			0.0							0.9
29		5.2	0.0	0.0		1.8		0.0		1.1	0.0	0.0		0.0	0.0	0.0	0.0									1.0
30	0.0	5.3	0.0	0.0	0.0					1.5	0.7	0.0	0.0	0.0	0.0	0.0		0.0								1.2
All	2.6	1.3	1.1	0.9	0.9	0.7	0.6	0.5	0.4	0.4	0.3	0.3	0.1	0.1	0.0	0.0	0.0	0.0	0.0	0.0	0.0	0.0	0.0	0.0	0.0	0.4

Percent of data fields changed

- 0.0
- 1.0
- 5.0
- 10.0

CRF ID	CRF Name	CRF ID	CRF Name
1	Early Drug Discontinuation	14	Randomization Result
2	Health History	15	Specimen Form
3	Discharge	16	Arm Exclusions
4	Study Drug Administration	17	Arm Exclusion & Contraindicated Medications
5	Off Study	18	IC14 / Narsoplimab Specimen Collection
6	Adverse Event	19	Study Drug Administration - Remdesivir
7	Daily Data, Day 29 Onward	20	Observational Patient Blood Draw
8	Death	21	Labs (OneSource)
9	Concomitant medications	22	Medications (OneSource)
10	Daily Data	23	Razuprotafib Monitoring: First Dose
11	Eligibility & Consent	24	Razuprotafib Monitoring: Subsequent Dose
12	Protocol Deviation	25	Study Drug Administration - Imatinib
13	ePRO Delivery Preference		

Supplementary Figure 5: Median posterior hazard ratios and 95% CI of arms before/after monitoring

A) recovery, before and after monitoring and B) death, before and after monitoring. CI = confidence intervals

A

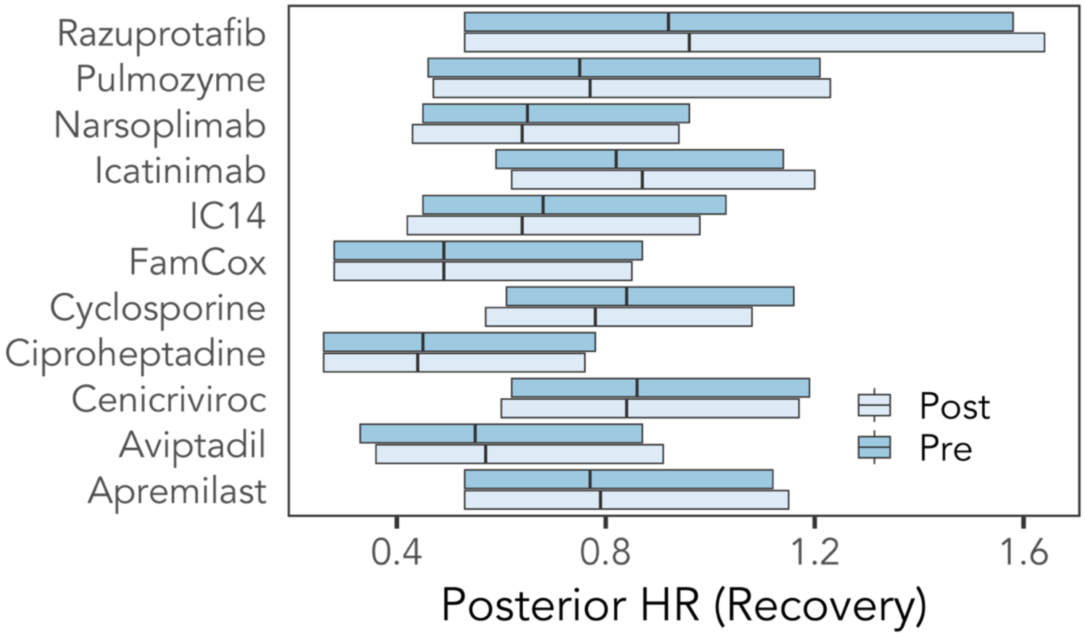

B

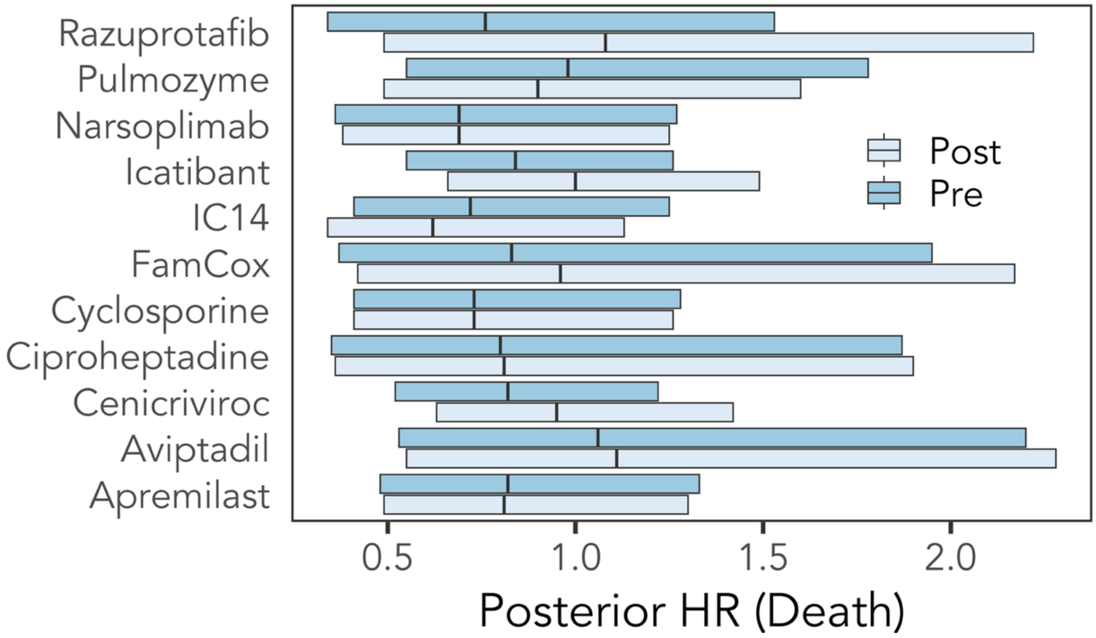

** It is important to note that other trial activities, including data entry and data cleaning from the sites, as well as activities of the DMC, SWG, and safety team, were ongoing during the period of retrospective monitoring. Therefore, changes to the recovery outcome pre- and post- monitoring may not be exclusively related to the retrospective monitoring; it is unclear whether or not any of these changes would have been missed in the absence of retrospective monitoring.*

3. Supplementary Tables

Supplementary Table 1: Electronic Case Report Forms utilized in the I-SPY COVID trial classified by risk level

eCRF	Medium or High Risk
Adverse Event	yes
Arm Exclusion & Contraindicated Medications	yes
Arm Exclusions	yes
Concomitant Medications	yes
Daily Data	yes
Daily Data, Day 29 Onward	yes
Death	yes
Discharge	no
Early Drug Discontinuation	no
Eligibility & Consent	yes
ePRO Delivery Preference	no
Health History	yes
IC14 / Narsoplimab Specimen Collection	no
Labs (OneSource)	no
Medications (OneSource)	no
Observational Patient Blood Draw	no
Off Study	no
Protocol Deviation	yes
Randomization Result	yes
Razuprotafib Monitoring: First Dose	yes
Razuprotafib Monitoring: Subsequent Dose	yes
Specimen Form	no
Study Drug Administration	yes
Study Drug Administration - Imatinib	no
Study Drug Administration - Remdesivir	yes

Supplementary Table 2: List of question on survey distributed to sites to estimate the cost of retrospective monitoring to sites.

Question	Text
1	How many staff and investigator man-hours in days do you estimate that your site spent IN TOTAL for remote site visits, monitoring visits, and audit visits? Include all time spent by your team preparing for visits in total. (For example 3 staff members each spending 4 hours would total 12 hours)
2	How much additional staff time do you estimate that your site spent IN TOTAL man hours as counted in days for in person site visits and audits? Include all time spent preparing for visits.(For example 3 staff members each spending 4 hours would total 12 hours)
3	How much additional staff time in man-hours do you estimate that your site spent IN TOTAL familiarizing/onboarding new monitors with your site, the study, and the EDC?(For example 3 staff members each spending 4 hours would total 12 hours)
4	How much additional staff time do you estimate on average that your site spent on average responding to and resolving EACH QUERY on Adverse Events?
5	How much additional staff time do you estimate that your site spent on average responding to and resolving EACH QUERY on Daily Data forms?
6	How much additional staff time do you estimate that your site spent on average responding to and resolving EACH QUERY on Eligibility and Randomization?
7	How much additional staff time do you estimate that your site spent on average responding to and resolving EACH QUERY on standard of care lab data entry?
8	How much additional staff time do you estimate that your site spent on average responding to and resolving EACH QUERY on Protocol Deviations?
9	How much additional staff time do you estimate on average that your site spent on average responding to and resolving EACH QUERY on Study Drug administration?
10	How much additional staff time do you estimate that your site spent on average responding to and resolving EACH QUERY on Study termination queries (includes queries on death, discharge, early discontinuation)?
11	How much additional staff time per query do you estimate that your site spent on average responding to and resolving each of the following types of monitoring queries on ePRO?
12	Are there additional comments or information that you would like to provide regarding the amount of time and effort it took for your team to respond to monitoring queries and monitoring in general through December 2022?

Supplementary Table 3: Data changes in the EDC by eCRF type between 6/1/2022-12/31/2022.

eCRF Type	Number of eCRFs SDV'd	Number of data fields in eCRFs	Number of data fields changed during SDV	Percent data fields changed during SDV
Adverse Event	136	15392	104	0.7
Arm Exclusion	74	153	0	0
Daily eCRF (includes daily checklist)	4517	225256	816	0.4
Death	78	1961	10	0.5
Discharge	185	634	7	1.1
Early Drug Discontinuation	31	228	6	2.6
Eligibility & Consent	314	7230	19	0.3
ePRO Delivery Preference	140	907	1	0.1
Health History	291	9989	133	1.3
Labs	203	2842	0	0
Medications	789	4788	17	0.4
Observational Patient Blood Draw	5	11	0	0
Off Study	29	109	1	0.9
Protocol Deviation	266	2721	7	0.3
Randomization Result	316	2764	3	0.1
Specimen Form	507	51978	3	0
Study Drug Administration	2164	11775	107	0.9
Study Drug Specific eCRFs	56	1794	0	0

** It is important to note that in addition to retrospective monitoring and SDV, other trial activities were ongoing, such as data cleaning by the sites, meetings of the SWG, and DMC, which means that not all data changes were due to monitoring.*

Supplementary Table 4: Distribution of patients enrolled across clinical sites

Sites in the trial including whether site used OneSource for electronic source data capture at any point in the trial, the number of patients, eCRFs, and data fields subject to monitoring, and the number and percent of data fields changed during the period of retrospective monitoring.

Site	*OneSource*	*Patients*	*eCRFs*	*Data Fields*	*Changes*	*Percent*
1	Yes	1	12	236	1	0.4%
2	Yes	1	15	550	0	0.0%
3	No	2	99	2,560	11	0.4%
4	No	2	85	3,322	29	0.9%
5	No	2	64	2,860	1	0.0%
6	No	3	44	1,798	3	0.2%
7	Yes	4	255	6,295	34	0.5%
8	Yes	5	190	5,223	53	1.0%
9	Yes	5	150	5,246	35	0.7%
10	Yes	6	114	2,239	0	0.0%
11	No	6	88	2,913	23	0.8%
12	No	6	127	4,645	8	0.2%
13	No	7	275	6,539	24	0.4%
14	No	7	167	7,465	13	0.2%
15	No	8	225	9,137	30	0.3%
16	No	8	168	8,578	14	0.2%
17	No	9	241	8,844	12	0.1%
18	No	9	690	12,218	94	0.8%
19	Yes	10	271	12,695	56	0.4%
20	No	13	361	14,036	127	0.9%
21	No	16	530	18,607	72	0.4%
22	No	17	313	12,365	149	1.2%
23	Yes	17	562	18,849	57	0.3%
24	No	18	446	16,401	9	0.1%
25	Yes	18	568	20,859	36	0.2%
26	No	20	361	10,519	53	0.5%
27	No	21	644	21,655	18	0.1%
28	Yes	24	712	26,336	140	0.5%
29	Yes	25	700	22,834	23	0.1%
30	Yes	43	1624	54,708	109	0.2%

Supplementary Table 5: Data changes in the EDC during the period of retrospective monitoring between 6/1/2022-12/31/2022

Stratified by OneSource status at that site, amongst sites that used OneSource. Difference between OneSource being operation and not being operational is significant (Chi-squared p=0.03)

OneSource operational	eCRFs	Data Fields	Changes	Percent Changed
No	8,948	309,038	1,142	0.37
Yes	1,153	31,494	92	0.29

Supplementary Table 6: Additional AE ECRFs completed during 6/1/2022-12/31/2022 by source and grade

	Grade					
**Source**	**1**	**2**	**3**	**4**	**5**	**Total**
Retrospective SDV	1	1	8	1	0	11
Safety Working Group/Safety Team	0	2	9	6	4	21
Site	1	3	2	1	1	8
Total	2	6	19	8	5	40

Supplementary Table 7: Calculation of hours used for monitoring by the sites

Query Type	Number of queries	Hours per query (from survey)	Total hours for query type
Adverse Evens	161	0.65	105
Other	50	0.75	37
Daily Data	2187	0.42	919
Randomization Eligibility	1331	0.23	306
ePRO	23	0.49	11
Lab	0	0.74	0
Protocol deviation	14	1.44	20
Drug	44	1.44	63
**All Queries**			**1461**
	**Sites**	**Hours per site (from survey)**	**Total Hours**
Onboarding Monitors	30	35.3	1058
In-Person Monitoring visits	30	40.4	1211
Remove Monitoring visits	30	40.4	1211
**Total time**		165	**4941**
