# Supplementary material for: Systematic data capture reduces the need for source data verification: exploratory analysis from a phase 2 multicenter randomized controlled platform trial
Source: Commun Med (Lond). 2025 Oct 29;5:444. doi: 10.1038/s43856-025-01126-9 (PMC12572212; doi:10.1038/s43856-025-01126-9)
Supplement: Supplementary file 2 — description of additional supplementary files [file 43856_2025_1126_MOESM2_ESM.pdf]

Description of Additional Supplementary Files

File name: Supplementary Data 1

Description: Data underlying Figure 2
