# Supplementary material for: Systematic data capture reduces the need for source data verification: exploratory analysis from a phase 2 multicenter randomized controlled platform trial
Source: Commun Med (Lond). 2025 Oct 29;5:444. doi: 10.1038/s43856-025-01126-9 (PMC12572212; doi:10.1038/s43856-025-01126-9)
Supplement: Supplementary file 4 — reporting summary [file 43856_2025_1126_MOESM4_ESM.pdf]

nature portfolio

Corresponding author(s): Laura J Esserman

Last updated by author(s): 2025.06.20

Reporting Summary

Nature Portfolio wishes to improve the reproducibility of the work that we publish. This form provides structure for consistency and transparency in reporting. For further information on Nature Portfolio policies, see our Editorial Policies and the Editorial Policy Checklist.

Statistics

For all statistical analyses, confirm that the following items are present in the figure legend, table legend, main text, or Methods section.

n/a	Confirmed	
☐	☒	The exact sample size (*n*) for each experimental group/condition, given as a discrete number and unit of measurement
☒	☐	A statement on whether measurements were taken from distinct samples or whether the same sample was measured repeatedly
☐	☒	The statistical test(s) used AND whether they are one- or two-sided<br>*Only common tests should be described solely by name; describe more complex techniques in the Methods section.*
☒	☐	A description of all covariates tested
☒	☐	A description of any assumptions or corrections, such as tests of normality and adjustment for multiple comparisons
☒	☐	A full description of the statistical parameters including central tendency (e.g. means) or other basic estimates (e.g. regression coefficient) AND variation (e.g. standard deviation) or associated estimates of uncertainty (e.g. confidence intervals)
☒	☐	For null hypothesis testing, the test statistic (e.g. *F*, *t*, *r*) with confidence intervals, effect sizes, degrees of freedom and *P* value noted<br>*Give P values as exact values whenever suitable.*
☒	☐	For Bayesian analysis, information on the choice of priors and Markov chain Monte Carlo settings
☒	☐	For hierarchical and complex designs, identification of the appropriate level for tests and full reporting of outcomes
☒	☐	Estimates of effect sizes (e.g. Cohen's *d*, Pearson's *r*), indicating how they were calculated

Our web collection on statistics for biologists contains articles on many of the points above.

Software and code

Policy information about availability of computer code

Data collection	Data was collected in OpenClinica EDC with the custom OneSource platform which is described in the manuscript
Data analysis	Data was tabulated and analyzed in microsoft Excel for Mac Version 16.97.2

For manuscripts utilizing custom algorithms or software that are central to the research but not yet described in published literature, software must be made available to editors and reviewers. We strongly encourage code deposition in a community repository (e.g. GitHub). See the Nature Portfolio guidelines for submitting code & software for further information.

Data

Policy information about availability of data

All manuscripts must include a data availability statement. This statement should provide the following information, where applicable:

- Accession codes, unique identifiers, or web links for publicly available datasets
- A description of any restrictions on data availability
- For clinical datasets or third party data, please ensure that the statement adheres to our policy

The data reported in this manuscript is maintained by the study sponsor, Quantum Leap Healthcare Collaborative (QLHC). Data can be shared with investigators external to the trial following approval by QLHC and the I-SPY COVID Data Access and Publications Committee, beginning at the time of publication of this article. This request can be initiated by contacting the corresponding author of this manuscript.

Research involving human participants, their data, or biological material

Policy information about studies with human participants or human data. See also policy information about sex, gender (identity/presentation), and sexual orientation and race, ethnicity and racism.

Reporting on sex and gender	N/A
Reporting on race, ethnicity, or other socially relevant groupings	N/A
Population characteristics	Here, we include results related to all 11 investigational agent arms and the control arm open between 7/30/2020 - 1/26/2022 (see Table 1), enrolling a total of 1,111 participants across 30 master sites, generating a total of 44,486 eCRFs.13
Recruitment	n/a
Ethics oversight	The trial included 42 clinical sites (30 master sites and 12 satellites) in the U.S. and was approved by a central IRB (Wake Forest University IRB, Winston-Salem, NC). I-SPY COVID was conducted in accordance with Good Clinical Practice guidelines and the Helsinki Declaration, and all participants or their surrogates provided informed consent prior to enrollment in the interventional arms of the study.

Note that full information on the approval of the study protocol must also be provided in the manuscript.

Field-specific reporting

Please select the one below that is the best fit for your research. If you are not sure, read the appropriate sections before making your selection.

☒ Life sciences ☐ Behavioural & social sciences ☐ Ecological, evolutionary & environmental sciences

For a reference copy of the document with all sections, see nature.com/documents/nr-reporting-summary-flat.pdf

Life sciences study design

All studies must disclose on these points even when the disclosure is negative.

Sample size	Based on available data
Data exclusions	No data was excluded
Replication	n/a
Randomization	n/a
Blinding	n/a

Reporting for specific materials, systems and methods

We require information from authors about some types of materials, experimental systems and methods used in many studies. Here, indicate whether each material, system or method listed is relevant to your study. If you are not sure if a list item applies to your research, read the appropriate section before selecting a response.

Materials & experimental systems

n/a	Involved in the study	
☒	☐	Antibodies
☒	☐	Eukaryotic cell lines
☒	☐	Palaeontology and archaeology
☒	☐	Animals and other organisms
☐	☒	Clinical data
☐	☐	Dual use research of concern
☒	☐	Plants

Methods

n/a	Involved in the study	
☒	☐	ChIP-seq
☒	☐	Flow cytometry
☒	☐	MRI-based neuroimaging

Clinical data

Policy information about clinical studies

All manuscripts should comply with the ICMJE guidelines for publication of clinical research and a completed CONSORT checklist must be included with all submissions.

Clinical trial registration

https://clinicaltrials.gov/study/NCT04488081

Study protocol

Files DC, Matthay MA, Calfee CS, et al. I-SPY COVID adaptive platform trial for COVID-19 acute respiratory failure: rationale, design and operations. BMJ Open. 2022;12(6):e060664. doi:10.1136/bmjopen-2021-060664

Data collection

Data capture in I-SPY COVID was optimized to minimize the burden on clinicians, while capturing essential data elements accurately and completely. The core data collection tool was the daily eCRF, which included details on disease severity, treatments received, concomitant medications, and a discrete set of labs to enable evaluation of all major organ systems. A key innovation was that the daily eCRF also included a short checklist of significant clinical events that were predefined by trial stakeholders (Figure 1 and Figure S1). The checklist required sites to assess daily whether the patient suffered adverse events common in critical illness due to COVID-19, such as a pneumothorax, pulmonary embolism, or initiation of antibiotics. The goal of this checklist was to limit reporting bias in this open-label trial, by capturing adverse event data systematically on all participants.

Outcomes

Number of data corrections, cost per data correction, number of conclusions changed

Plants

Seed stocks

Report on the source of all seed stocks or other plant material used. If applicable, state the seed stock centre and catalogue number. If plant specimens were collected from the field, describe the collection location, date and sampling procedures.

Novel plant genotypes

Describe the methods by which all novel plant genotypes were produced. This includes those generated by transgenic approaches, gene editing, chemical/radiation-based mutagenesis and hybridization. For transgenic lines, describe the transformation method, the number of independent lines analyzed and the generation upon which experiments were performed. For gene-edited lines, describe the editor used, the endogenous sequence targeted for editing, the targeting guide RNA sequence (if applicable) and how the editor was applied.

Authentication

Describe any authentication procedures for each seed stock used or novel genotype generated. Describe any experiments used to assess the effect of a mutation and, where applicable, how potential secondary effects (e.g. second site T-DNA insertions, mosiacism, off-target gene editing) were examined.
